# Pharmacological Insights Into Safety and Efficacy Determinants for the Development of Adenosine Receptor Biased Agonists in the Treatment of Heart Failure

**DOI:** 10.3389/fphar.2021.628060

**Published:** 2021-03-11

**Authors:** Patricia Rueda, Jon Merlin, Stefano Chimenti, Michel Feletou, Jerome Paysant, Paul J. White, Arthur Christopoulos, Patrick M. Sexton, Roger J. Summers, William N. Charman, Lauren T. May, Christopher J. Langmead

**Affiliations:** ^1^ Drug Discovery Biology, Monash Institute of Pharmaceutical Sciences, Monash University, Parkville, VIC, Australia; ^2^ Cardiovascular Discovery Research Unit, Institut de Recherches Servier, Suresnes, France

**Keywords:** adenosine A1 receptor agonist, heart failure, G protein-coupled receptor, Biased agonism, neladenoson, capadenoson, VCP-746

## Abstract

Adenosine A_1_ receptors (A_1_R) are a potential target for cardiac injury treatment due to their cardioprotective/antihypertrophic actions, but drug development has been hampered by on-target side effects such as bradycardia and altered renal hemodynamics. Biased agonism has emerged as an attractive mechanism for A_1_R-mediated cardioprotection that is haemodynamically safe. Here we investigate the pre-clinical pharmacology, efficacy and side-effect profile of the A_1_R agonist neladenoson, shown to be safe but ineffective in phase IIb trials for the treatment of heart failure. We compare this agent with the well-characterized, pan-adenosine receptor (AR) agonist NECA, capadenoson, and the A_1_R biased agonist VCP746, previously shown to be safe and cardioprotective in pre-clinical models of heart failure. We show that like VCP746, neladenoson is biased away from Ca^2+^ influx relative to NECA and the cAMP pathway at the A_1_R, a profile predictive of a lack of adenosine-like side effects. Additionally, neladenoson was also biased away from the MAPK pathway at the A_1_R. In contrast to VCP746, which displays more ‘adenosine-like’ signaling at the A_2B_R, neladenoson was a highly selective A_1_R agonist, with biased, weak agonism at the A_2B_R. Together these results show that unwanted hemodynamic effects of A_1_R agonists can be avoided by compounds biased away from Ca^2+^ influx relative to cAMP, relative to NECA. The failure of neladenoson to reach primary endpoints in clinical trials suggests that A_1_R-mediated cAMP inhibition may be a poor indicator of effectiveness in chronic heart failure. This study provides additional information that can aid future screening and/or design of improved AR agonists that are safe and efficacious in treating heart failure in patients.

## Introduction

Heart failure (HF) covers a wide range of clinical and pathophysiological conditions. It is broadly defined as a clinical syndrome whereby the heart fails to supply enough blood to fulfill the metabolic needs of the tissues (Coronel et al., 2001). In general, the pathophysiology of HF is described by two major categories: 1) HF with reduced ejection fraction (HFrEF), where the left ventricular ejection fraction (LVEF) is <40%; and 2) HF with preserved ejection fraction (HFpEF) where LVEF >50%. A third recently described category of HF with mid-range ejection fraction (HFmEF) is still controversial, but identifies patients with a LVEF of 40–49% in patients with different underlying characteristics and pathophysiology (Ponikowski et al., 2016). There is a forecast 46% increase in HF prevalence by 2030, with over eight million cases in the US alone (Heidenreich et al., 2013; Mozaffarian et al., 2015) illustrating the need for effective treatments.

The most recent guidelines for HFrEF treatment includes angiotensin receptor-neprilysin inhibitors (ARNI) (e.g. sacubitril/valsartan), which reduce the effect of maladaptive neurohormones and block cardiac remodeling (Jessup et al., 2016). Although both basic research and the establishment of clear evidence-based clinical guidelines is improving management of HFrEF, these therapies have adverse hemodynamic effects (Vaduganathan et al., 2015). There is still a need for an approved pharmacological intervention for the treatment of HFpEF (Bonsu et al., 2018) underpinned by an increase in prevalence of this condition (Owan et al., 2006).

To address this, it has been suggested that therapeutic strategies should be aimed at directly attenuating adverse cardiac remodeling whilst being haemodynamically neutral (Vaduganathan et al., 2015). As previously suggested (Greene et al., 2016), fine-tuned modulation of adenosine receptors (ARs), and in particular the A_1_ subtype (A_1_R), may provide a route to fulfill these criteria, with the potential to be haemodynamically neutral, improve cardiomyocyte (CM) energetics, cardiac structure and function, and prevent further tissue injury by inducing cardioprotection and reduction of interstitial fibrosis.

Adenosine has been long-known to exert pleiotropic protective and regenerative effects and activates all four adenosine receptor (AR) subtypes (A_1_, A_2A_, A_2B_ and A_3_Rs) in different tissues (Linden, 2005). ARs are G protein-coupled receptors (GPCRs) originally classified by their pharmacological response to adenosine, with A_1_ and A_3_R inhibiting, and A_2A_ and A_2B_R activating adenylate cyclase (Fredholm et al., 2011). The adenosinergic system impacts major aspects of cardiovascular function, including beat rate, conduction, autonomic control, perfusion, growth and remodeling, and ultimately protection to injury (Headrick et al., 2013). It is well established that all four receptor subtypes are expressed in the cardiovascular system and that their expression levels alter following injury (Cabiati et al., 2014), although full characterization of cell-specific and relative subtype abundance of AR expression is unknown.

In the setting of heart failure, ARs modulate adaptive and maladaptive responses. Cardiomyocyte hypertrophy plays an important role in this process and neurohormonal factors such as catecholamines, angiotensin II or endothelin are involved in cardiac hypertrophy and failure (Frey and Olson, 2003). Inflammation is also a hallmark of cardiac hypertrophy and involves factors such as interleukin (IL)-1β or tumor necrosis factor-alpha (TNF-α) (Erten et al., 2005; Kuusisto et al., 2012). Importantly, A_1_R agonists reduce both neurohumoral- and inflammation-driven hypertrophy (Liao et al., 2011; Chuo et al., 2016; Puhl et al., 2016).

Cardiac fibroblasts also contribute to the heart failure phenotype, as adverse remodeling by these cells leads to excess generation of extracellular matrix, fibrosis, and causes contractile dysfunction. The A_2B_R, which is highly expressed in fibroblasts (Epperson et al., 2009), is the main AR subtype involved in cardiac fibroblast proliferation and collagen synthesis (Dubey et al., 1997; Dubey et al., 1998; Dubey et al., 2001; Chen et al., 2004).

Despite the preclinical efficacy shown by AR agonists, further development of these agents has been compromised by the widespread expression of ARs throughout the body, and their pleiotropic effects on the cardiovascular system. These on-target side effects include modulation of blood pressure, heart rate, atrioventricular (AV) conduction, and renal function. The A_1_R (highly expressed in the atria) is responsible for changes in heart rate and conduction (Belardinelli et al., 1995; Yang et al., 2007), while the A_2A_R and A_2B_R subtypes (found in smooth muscle and endothelium) play major roles in vasoregulation (Kemp and Cocks, 1999; Sato et al., 2005). Accordingly, activation of these receptors often leads to changes in blood pressure. Furthermore, AR activation plays an important role in the hemodynamic balance of the kidney, as A_1_R mediate cortical vasoconstriction and A_2A_R/A_2B_R mediate medullar vasodilation, thus reducing filtration fraction (Vallon et al., 2008). This is an important consideration where patients with HF exhibit abnormal cardiorenal hemodynamics that ultimately exacerbate the disease (Damman and Testani, 2015).

In addition to ligands that control the strength of signaling of a GPCR (efficacy), receptors are highly dynamic proteins with different active-state conformations that can be linked to different cellular outcomes. By extension, ligands stabilizing different conformations can specifically promote a subset of signaling or regulatory pathways, a phenomenon known as biased agonism (Kenakin and Christopoulos, 2013). This affords the potential to target GPCRs with improved on-target specificity, as recently established in preclinical models for several GPCR agonists (DeWire et al., 2013; Brust et al., 2015; Baltos et al., 2016; Mallipeddi et al., 2017; Mores et al., 2019). Notably, amongst A_1_R agonists, VCP746 (and derivatives) are biased away from intracellular calcium mobilization relative to other pathways and yield more therapeutically favourable *ex vivo* pharmacology (Valant et al., 2014; Baltos et al., 2016).

Collectively these data suggest the possibility of identifying adenosine receptor agonists with bias profiles that yield efficacy with high therapeutic index. In 2012 Bayer described capadenoson as a non-ribose, high affinity, highly selective A_1_R agonist with good pharmacokinetics, efficacy, and a promising safety profile. It displayed reduced bradycardia in preclinical models and no effects on heart rate at rest in clinical studies (clinical study NCT00568945), while maintaining full cardioprotective potential with amelioration of markers of structural remodeling in preclinical models (Albrecht-Küpper et al., 2012; Sabbah et al., 2013). However, CNS safety and low solubility limited its utility and prompted the development of an improved agent in the form of neladenoson.

Neladenoson (BAY 1067197) is a pro-drug of the pharmacologically active moiety, reportedly a partial A_1_R agonist, that therefore addresses some of the limitations presented by capadenoson. Neladenoson was cardioprotective in rodents while showing fewer central side effects (Meibom et al., 2017), and was safe and well tolerated in both phase I and phase IIa clinical studies (Voors et al., 2017). Despite this promising preclinical and clinical safety profile, in two phase IIb clinical trials in HFrEF and HFpEF patients (PANTHEON and PANACHE, respectively), neladenoson failed to meet its primary and secondary endpoints for efficacy (Bertero and Maack, 2019; Shah et al., 2019; Voors et al., 2019).

Herein we sought to compare the pre-clinical pharmacology of neladenoson, capadenoson, the tool A_1_R biased agonist VCP746, and the pan-AR agonist, NECA, in molecular signaling assays across adenosine receptors, and in *in vivo* and *ex vivo* models of cardio-renal function. Neladenoson displayed high selectivity for the A_1_R, and presented a bias profile similar to VCP746 that is predictive of a lack of overt adenosine-like side effects. However, there was some divergence in other aspects of biased signaling and adenosine receptor subtype activity that might help in the design of future agents that are not only safe, but which have efficacy in treating heart failure in patients.

## Materials and Methods

### Materials

Research reagents were obtained from the following suppliers: Dulbeco’s modified Eagle medium (DMEM; Life Technologies Australia, 11965118), Hanks’ Balanced Salts (Sigma-Aldrich, H2387), trypsin (Life Technologies Australia, 15090046), antibiotic/antimycotic (Life Technologies Australia, 15240062), penicillin/streptomycin (Gibco, 15140-122), FBS (Gibco), Adenosine deaminase (ADA; Sigma-Aldrich, 10102105001), hygromycin B (Scientific INC., H-1012-PBS), Probenecid (Sigma-Aldrich, P8761), Rolipram (Sigma-Aldrich, R6520), Hoechst33342 (ThermoFisher Scientific, H3570), Propidium iodide (Sigma-Aldrich, P4170), 5-Bromo-2′-deoxyuridine (BrdU; Sigma-Aldrich, B5002), 5’-(N-ethylcarboxamido), 5′-N-ethylcarboxamidoadenosine (NECA; Sigma-Aldrich, E2387), SLV-320 (Tocris, RDS334410), CGS21680 (Tocris, 1063; Sigma-Aldrich, C141), BAY60-6583 (Tocris, 4472), 2′Me-CCPA (Tocris, 2281), MRS1754 (Tocris, 2752), methoxamine (Sigma-Aldrich, M6524), Angiotensin II (AngII; Sigma-Aldrich A9525), interleukin 1beta (IL-1β; R&D systems, 201-LB-005), tumor necrosis factor alpha (TNFα; R&D systems, 210-TA), Lactate dehydrogenase (LDH) Activity Assay Kit (Sigma-Aldrich, MAK066), Adenosine Triphosphate (ATP; Sigma-Aldrich, A26209), Forskolin (Sigma-Aldrich, F3917), Fluo-4 AM (Invitrogen, F14201), Pertussis toxin (PTX; Sigma-Aldrich, P7208), [^3^H]-Leucine (Perkin Elmer, NET135H001MC), [^3^H]-Proline (Perkin Elmer, NET483001MC), Lance cAMP detection kit (PerkinElmer, AD0262) Alphascreen Surefire ERK1/2 (Thr202/Tyr204) Phosphorylation kit (PerkinElmer, TGRESB), Alphascreen Surefire Akt1/2/3 (p-Ser473) Phosphorylation kit (PerkinElmer, TGRA4S), collagenase Type II (Scimar Australia, LS004176). Neladenoson (as the active metabolite) and capadenoson were synthesized by Servier, and VCP746 was made by SYNthesis Pty. (Melbourne, Australia).

### Cell Culture

Flp-IN CHO-A_1_R, -A_2A_R, -A_2B_R, and -A_3_R stable cell lines were generated as previously described (Stewart et al., 2009; Vecchio et al., 2016b), maintained in DMEM supplemented with 10% FBS and 500 μg/ml hygromycin B and confirmed mycoplasma-free.

### cAMP Accumulation

Cells were trypsinized and seeded in DMEM with 10% FBS in 96-well plates at 20,000 cells/well and incubated overnight. Cells were then washed and incubated in cAMP stimulation buffer (140 mM NaCl, 5 mM KCl, 0.8 μM MgSO_4_, 0.2 mM Na_2_HPO_4_, 0.44 mM KH_2_PO_4_, 1.3 mM CaCl_2_, 5.6 mM d-glucose, 5 mM HEPES) containing ADA (0.1 U/ml), rolipram (10 μM) and BSA (0.1%) at 37°C in a humidified incubator with 5% CO_2_ for 1 h. Compounds were then added and incubated for 30 min. When Gα_i_-mediated signaling was evaluated, 3 μM forskolin was added to the cells 10 min after compound addition. Stimulation was terminated by removal of buffer and replacement with ice-cold 100% ethanol. After ethanol evaporation, cells were lyzed in lysis buffer and cAMP levels were detected using the Lance cAMP kit following manufacturer’s instructions. cAMP levels were extrapolated using the standard provided in the kit and then normalized to the forskolin control.

### Calcium Mobilization

Cells were trypsinized and seeded in DMEM with 10% FBS in 96-well plates at 40,000 cells/well for 8 h at 37 °C in a humidified incubator with 5% CO_2_. Cells were then incubated in serum-free medium overnight, washed and incubated in calcium stimulation buffer (146 mM NaCl, 5 mM KCl, 1 mM MgSO_4_, 1.3 mM CaCl_2_, and 1.5 mM NaHCO_3_, 10 mM d-glucose, 10 mM HEPES) containing ADA (0.1 U/ml), probenecid (2.5 mM), BSA (0.5%) and Fluo-4 AM (1 μM) for 1 h. Fluorescence was detected on a FlexStation plate reader (molecular Devices; Sunnyvale, CA, USA) after the automated addition of buffer in the absence or presence of receptor ligands. Data were analyzed as the difference between the peak and baseline reads and normalized to the ATP (100 μM) response.

### ERK1/2 and Akt1/2/3 Phosphorylation

Cells were trypsinized and seeded in DMEM with 10% FBS in 96-well plates at 40,000 cells/well for 8 h at 37°C in a humidified incubator with 5% CO_2_. Cells were then incubated in serum-free medium overnight, and ADA (0.1 U/ml) added 1 h prior to assay. Cells were then exposed to DMEM in the absence or presence of receptor ligands and agonist concentration-response curves were generated at the time of peak response. Stimulation was terminated by rapid removal of media and addition of 50 μL/well or AlphaScreen SureFire kit lysis buffer. Detection of either ERK1/2 or Akt1/2/3 phosphorylation was performed as described in the corresponding AlphaScreen SureFire kits and fluorescence measured with an EnVision plate reader (PerkinElmer, Boston, MA). Data were normalized to the response elicited upon stimulation of cells with 10% FBS.

### Cell Survival

Cells were trypsinized and seeded in DMEM 10% plus FBS in 96-well plates at 40,000 cells/well for 8 h at 37 °C in a humidified incubator with 5% CO_2_. After 8 h, plates were rinsed in serum-free DMEM and then incubated in serum-free medium overnight. Media was then changed to fresh, sterile calcium stimulation buffer, containing ADA (0.1 U/mL) and pen/strep (1 U/mL) and incubated for a further 24 h. Hoechst33342 (200 μM; to define all cells) and propidium iodide (PI; 50 μg/ml; to define dead/dying cells) were added to two wells, incubated at 37°C for 30 min, and cell nuclei counts detected using the Operetta (PerkinElmer, Boston, MA) using manufacturer’s protocols. This defined 0% cell death for subsequent assay. Varying concentrations of adenosine receptor agonists were added to the remaining wells and cells incubated at 37°C for 24 h. Finally, Hoechst33342 and PI stains were added to all wells, incubated for 30 min, and nuclei counts detected on the Operetta. Immediately prior to addition of stains, buffer was removed from two wells and MilliQ water added to lyse cells as a positive control. Data are expressed as a percentage of surviving cells.

### Cardiomyocyte Isolation and Culture

Neonatal cardiac myocytes (CM) were isolated from 1 to 2 day-old Sprague-Dawley rat pups using enzymatic digestion. Briefly, animals were euthanized and the hearts isolated by thoracic incision and kept in Hanks solution. Then ventricles were isolated and incised at the apex to increase tissue surface exposure. Tissue was then incubated overnight at 4°C with trypsin. After trypsin deactivation with fresh FBS-supplemented DMEM, tissue was further digested by four cycles of collagenase incubation. Cells were then recovered by centrifugation and seeded in DMEM supplemented with FBS on gelatin-coated dishes for 2 h in order to select against adherent fibroblasts. The remaining floating cardiomyocytes where then collected, counted, and seeded in either 12-well plates at a density of 300,000 cells/well, or 96-well “chimney well” cell culture plates (Eppendorf) at a density of 37,500 cells/well. Cells were maintained in DMEM supplemented with 10% FBS and BrdU (100 μM) was included for the first three days of culture.

### [^3^H]-Leucine Incorporation in Cardiomyocytes

To measure hypertrophy by [^3^H]-leucine incorporation, after five days in culture cardiomyocytes were starved overnight in serum-free DMEM and then pre-treated with adenosine receptor agonists or vehicle for 2 h before hypertrophic stimuli [IL-1β (10 ng/ml), TNF-α (10 ng/ml), or Ang II (100 nM)], after which 1 μCi of [^3^H]-leucine was added to each well. Cells were incubated at 37°C with 5% CO_2_ in a humidified incubator for 72 h, washed with PBS and lyzed using 0.2 M NaOH. After adding UltimaGold scintilliant to the samples, radioactivity was detected using a MicroBeta2 Plate Counter (PerkinElmer Life Sciences). Data were normalized to the signal obtained for the vehicle treated samples.

### [^3^H]-Proline Incorporation in Cardiac Fibroblasts

Cardiac fibroblasts were recovered from gelatin-coated dishes described above through trypsination and plated in 12-well plates at a density of 50,000 cells/well for [^3^H]-proline incorporation assays in high glucose DMEM supplemented with 10% FBS. After 4 days cardiac fibroblasts were serum starved in DMEM overnight and then treated with vehicle, VCP746 or neladenoson 2 h prior to fibrotic stimuli TGFβ (10 ng/ml) or AngII (10 nM). [^3^H]-Proline (1 μCi/well) was then added to each well. After 72 h cells were washed, lyzed and radioactivity detected as per [^3^H]-leucine assay, described above.

### Cardiomyocyte Lactate dehydrogenase Release and PI Staining

To determine apoptotic effects of adenosine receptor agonists, a combined LDH release and PI stain assay was performed with an identical treatment regimen to [^3^H]-leucine incorporation assays: 2 h pre-treatment with adenosine receptor agonists before stimulation with IL-1β (10 ng/ml), TNF-α (10 ng/ml) or Ang II (100 nM) for 72 h at 37°C with 5% CO_2_ in a humidified incubator. On the day of assay, 20 μL of extracellular media from cardiomyocyte plates was transferred to a new 96-well plate, with NADH standard dilutions, and a colorimetric LDH release assay performed as per manufacturer’s instructions (Sigma-Aldrich; cat no: MAK066). Absorbance (450 nm) was detected at 3 min intervals (FlexStation) until it reached the upper range of standards. Data (“cell viability”) are expressed as a percentage of control-treated wells. In parallel, the cells without washing were treated with DMEM containing Hoescht33342 (200 μM) and PI (50 μg/ml) for 1 h at 37°C. Hoechst33342-and PI-positive nuclei were quantified on the Operetta using manufacturer’s protocol. Data (“cell survival”) are a ratio of PI/Hoechst33342 counts, expressed as a percentage of control-treated wells.

### Measurement of Beat Rate in Cardiomyocytes

Rat cardiomyocytes were cultured 4 days in 96-well plates before cells were checked visually to have widespread cell-cell contact and uniform beating across the well. Media was then changed to fresh DMEM with 10% FBS 24 h prior to assay, and 0.1U/mL ADA added 2 h prior to assay. For assay, spontaneous contractions were brightfield recorded (Nikon Ti-E microscope; 37°C, 5% CO_2_) for 100 frames at 10 frames/sec to define a basal beat rate. Cells were then incubated with ligand for 5 min and recorded again. Quantification of contractions was determined by time-resolved analysis (Image J 1.51n) of peak intensity of several representative cardiomyocytes within the field of view. All data are expressed as % of beat rate prior to addition for each replicate. Where SLV320 (1 μM) was used, it was added 15 min prior to basal recording.

### Measurement of Beat Rate in Isolated Rat Atria

Experiments were carried out on male Wistar rats (400-450 g) from JANVIER Labs (Center d’Elevage René JANVIER, Le Genest Saint-Isle, France). Rats were anesthetized with sodium pentobarbital (54.7 mg/kg intraperitoneal). Right atria were carefully removed and immediately immersed in physiological salt solution (PSS) at 4°C containing (in mM): NaCl 112, KCl 5, KH_2_PO_4_ 1, MgSO_4_ 1.2, CaCl_2_ 2.5, NaHCO_3_ 29.8, glucose 11.5, EDTA 0.02, pH 7.4 ± 0.05. Preparations were suspended vertically in an organ bath filled with 20 ml of PSS maintained at 35°C and gassed with a mixture of 95% O_2_ + 5% CO_2_. Isometric tension was recorded by means of a force transducer (EMKA Technologies, Paris, France). Atria were stretched to obtain a resting tension of 0.2–1 g. Right atria spontaneous beating frequency was measured using specific software (IOX EMKA Technologies, Paris, France). Only preparations with a basal beating rate between 200 and 300 beats per minute (bpm) were included.

After a 30 min equilibration period, dose-response curves were generated with cumulative concentrations (10^−9^ M to 10^−5^ M) of NECA, CGS21680, BAY60-6583 or 2′MeCCPA every 30 min. Due to their long kinetic of effect, VCP746 and neladenoson were tested with a single concentration (10^−7^ M to 10^−5^ M) per preparation, beating rate was measured at 120 min after each concentration. To assess NECA specificity, atria were pre-treated (30 min) with the specific A1R antagonist SLV320 at 10 nM or 100 nM. Then, two successive concentrations of NECA (10 and 100 nM) were added for 1 h.

Spontaneous beating frequency of right atria, measured at fixed time or concentration, was expressed in bpm. The effects of the compounds were expressed as percent changes from the basal atrial beating frequency.

### Measurement of Heart Rate in Moving, Conscious Animals

Male Wistar rats (8 weeks old, n = 4–7) were implanted with a radio telemetric device equipped with a pressure transducer (HDS10, Data Sciences International) under anesthesia with isoflurane (2%, VETFLURANE^®^, VIRBAC, France). Rats received buprenorphine (50 μg/kg s. c., BUPRECARE^®^, Axience SAS, France) for analgesia prior to surgery. After laparotomy the telemetric device catheter was inserted into the abdominal aorta and secured with cellulose patch and tissue adhesive (3 M, VETBOND™) around the insertion point. The body of the device was placed in the abdominal cavity and sutured to the inner side of the abdominal musculature then the skin plan was closed.

After a recovery period of two weeks a catheter (polyethylene/silastic) was introduced in jugular vein for drug administration by infusion. Briefly, the jugular vein was dissected and a small incision was made in order to introduce the catheter into the vessel. The catheter was tunneled subcutaneously to the dorsum of the neck and drawn back up through the skin. Rats were kept on a heating pad (38°C) until fully recovered from anesthesia. Animals were allowed 2-3 days recovery before being used for the experiment. On the day of study, 20 min infusion protocols were performed at different doses of test compounds to achieve predetermined plasma drug concentrations. Acquisition of heart rate was done with IOX (EMKA, France), sampled and averaged over 5s every 5s. The mean of the values recorded for each dose was calculated using the software Data Analyst (EMKA, France).

### Vasorelaxation of Rat Thoracic Aorta

Experiments were carried out on male Wistar rats (400–450 g) from JANVIER Labs (Center d’Elevage René JANVIER, Le Genest Saint-Isle, France). Rats were anesthetized with sodium pentobarbital (54.7 mg/kg intraperitoneal). The thoracic aorta was quickly removed placed in ice-cold physiological salt solution (PSS) at 4°C containing (in mM): NaCl 112, KCl 5, KH_2_PO_4_ 1, MgSO_4_ 1.2, CaCl_2_ 2.5, NaHCO_3_ 29.8, glucose 11.5, EDTA 0.02, pH 7.4 ± 0.05. Then the aorta was cleaned of adhering fat and connective tissue and cut transversely into 3–4 mm rings denuded or not of endothelium and placed in 20 ml organ baths containing PSS maintained at 37°C with continuous bubbling of 95% O_2_ + 5% CO_2_. Aortic rings were mounted vertically between two stainless wire hooks and then suspended. For isometric force response measurement, the changes in tension of pre-contracted intact or denuded rings were continuously monitored and recorded using specific software (IOX EMKA Technologies, Paris, France). Aortic rings were equilibrated for 60 min with a resting force of 2.5 g.

Aortic rings were constricted with phenylephrine (1 µM) to obtain a steady contraction then relaxed with cumulative acetylcholine concentration (0.01–10 µM) to check the integrity of the endothelium. The absence of endothelium was confirmed by the lack of responsiveness to acetylcholine. Aortic rings were washed several times with PSS and equilibrated for 30 min. Then the rings were constricted with phenylephrine (1 µM) to obtain a steady contraction and the agonists were added in cumulated concentrations (1 nM–10 µM) to the organ bath. At the end of each experiment, the rings were tested for viability by being maximally dilated with 100 µM papaverine. Relaxation was expressed as a percent of maximal relaxation to papaverine and as percent changes from phenylephrine contraction.

### Renal Vasodilation and Vasoconstriction

Male Wistar rats (300–400 g), purchased from Janvier Labs (Center d’Elevage René Janvier, Le Genest Saint-Isle, France) were anesthetized intraperitoneal with a mixture of ketamine (110 mg/kg) and xylazine (7.5 mg/kg). The left kidney was exposed by midline ventral laparotomy and the left renal artery was cannulated. The kidney was then perfused at constant flow via a peristaltic pump with warmed (37°C) and oxygenated (95% O_2_–5% CO_2_) Tyrode solution of the following composition (mM): NaCl 137; KCl 2.7; CaCl_2_ 1.8; MgCl_2_ 1.1; NaHCO_3_ 12.0; NaHPO_4_ 0.42; calcium disodium EDTA 0.026 and glucose 5.6. The perfused kidney was removed from the surrounding fat and placed in a perfusion chamber. The change in renal vascular resistance was recorded as changes in renal perfusion pressure (RPP) measured downstream of the pump via a pressure transducer (P10EZ, Statham, France) connected to a data acquisition system (IOX2, EMKA Technologies, France). Pharmacological agents were administered either via an infusion pump placed upstream to the perfusion pump (concentrations expressed below in mol/L) or injected as a bolus of 20 µL (doses expressed in mol) into the perfusion circuit.

In order to study renal vasodilator response, a sustained and stable vasoconstriction was maintained by permanent infusion of methoxamine (10 μmol/L). After stable vasoconstriction, dose-response curves were performed by using BAY60-6583 (1 ρmol to 10 nmol), neladenoson (10 ρmol to 30 nmol), or VCP746 (1 ρmol to 10 nmol). To verify the effect of BAY60-6583 in the renovascular responses to vasodilation, selective A2A (SCH 442416 100 nM) or A2B (MRS 1754 100 nM) receptor antagonists were administered by perfusion to the kidney 30 min before the agonist. Vasodilation was expressed by the negative delta between the response and Methoxamine constriction.

In order to measure renal vasoconstriction, methoxamine-treated (0.01 μmol/L) kidneys were stimulated with 0.1 μmol/L forskolin. Under these conditions, purine inhibition of adenylate cyclase would blunt the forskolin effect thereby producing vasoconstriction (Kenakin and Pike, 1987). Then 2′MeCCPA (1 ρmol to 10 nmol), NECA (1 ρmol to 100 nmol), neladenoson (1 ρmol to 30 nmol) or VCP746 (1 ρmol to 10 nmol) were administered by bolus injection to induce vasoconstriction. Before methoxamine and forskolin infusion two bolus injections of noradrenaline (NA, 0.3 nmol) were performed to obtain reference vasoconstriction. To verify 2′MeCCPA effect on renovascular response, a selective A1 receptors antagonist (SLV320 0.1 and 0.01 µM), was administered 30 min prior agonist administration. Vasoconstriction was expressed as difference between the response and Methoxamine/Forskolin induced constriction, or as the % of NA constriction for the experiment with 2′MECCPA.

### Statistics

All data are expressed as mean ± SEM or mean ± SD (as indicated) of *n* experiments. For concentration response data, curves were analyzed using three-parameter nonlinear curve fitting (GraphPad Prism 8.02) of grouped data. pK_b_ values of VCP746 at hA_3_ receptor were determined using the Schild method (Arunlakshana and Schild, 1959) and calculated using the operational model plus agonism (GraphPad Prism 8.02). Statistical analysis was performed using paired Student’s t-test, repeated measures one-way analysis of variance (ANOVA) or two-way ANOVA with Dunnett’s post-hoc test, as indicated in results. *p* < 0.05 was considered significant.

Ligand bias at adenosine receptors was analyzed using methods described previously (Kenakin and Christopoulos, 2013; Van Der Westhuizen et al., 2014). Cyclic AMP accumulation, calcium mobilization and protein phosphorylation assays were performed using all four AR agonists in parallel, allowing calculation of Log (τ/K_A_) values for each individual *n*, and subsequent calculation of bias to include *weighted* mean ± SEM of *n*. Bias (Log(τ/K_A_), ΔLog(τ/K_A_), ΔΔLog(τ/K_A_)) was calculated in Microsoft Excel (2010) and bias plots generated using the radar plot feature. Analysis of ΔLog(τ/K_A_) and ΔΔLog(τ/K_A_) were performed using multiple two-way ANOVA with multiple comparisons (GraphPad Prism 8.02).

### Study Approval

Animal experiments were conducted in accordance to either Servier Ethical committee guidelines or the Monash Institute of Pharmaceutical Sciences animal ethics committee-approved protocols (ethics approval number: MIPS.2017.18) and conformed to the requirements of the National Health and Medical Research Council of Australia Code of practice for the care and use of animals for scientific purposes.

## Results

### Neladenoson and Capadenoson Are Differentially Biased at the Adenosine A1 Receptor Compared With VCP746

**FIGURE 1:**
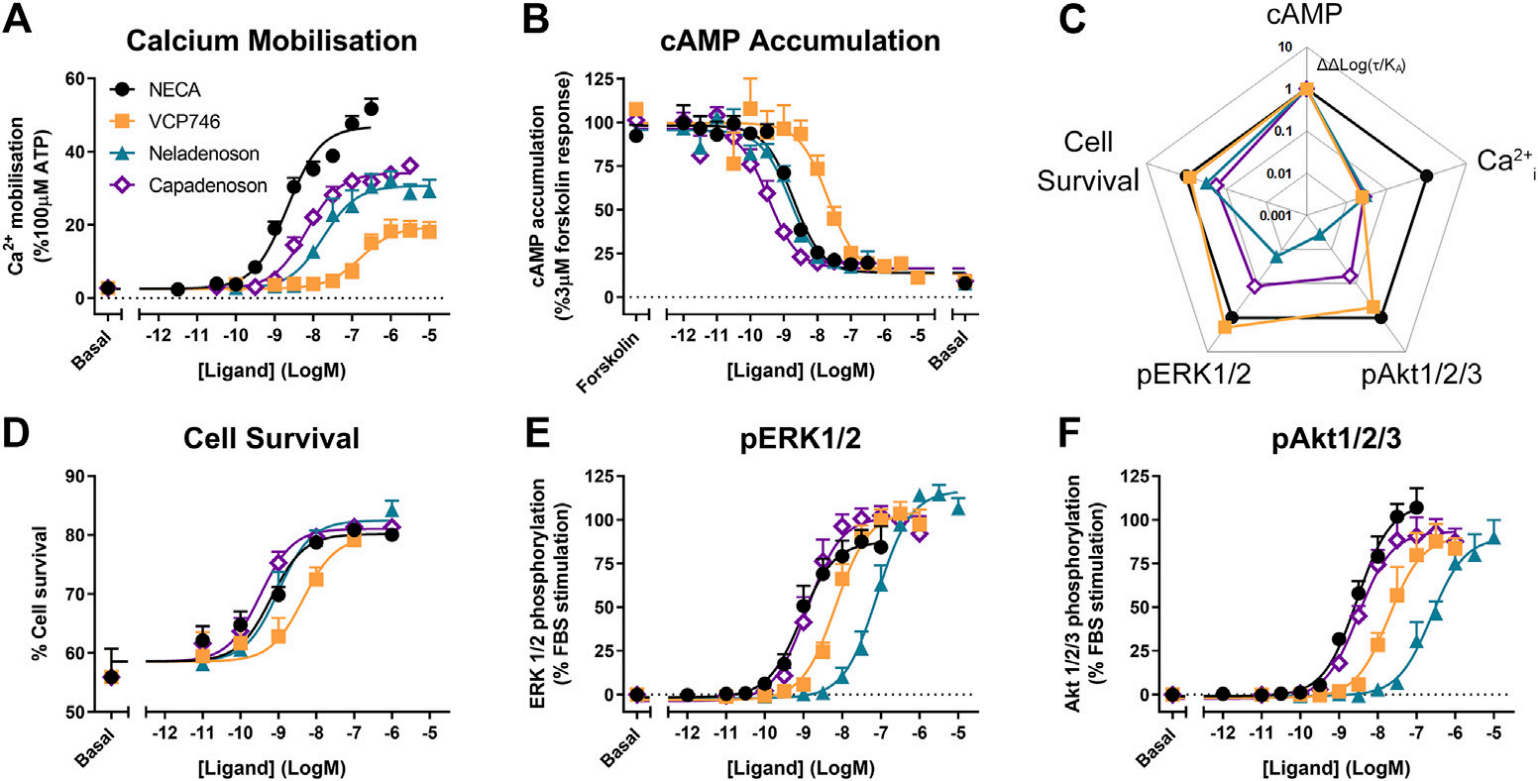
Multi-pathway profiling at the A_1_R. Adenosine receptor agonists were analyzed in CHO-hA_1_R cells in assays of calcium mobilization (**A**; n = 4), inhibition of cAMP accumulation (**B**; n = 6), cell survival (**D**; n = 3) or phosphorylation of ERK1/2 (**E**; n = 5) and Akt1/2/3 (**F**; n = 3). These data were used to calculate bias, where Log(τ/K_A_) are normalized to NECA (ΔLog(τ/K_A_)) and cAMP accumulation (ΔΔLog(τ/K_A_); **C**). Statistical significance of bias is reported in Supplementary Figure S1. Concentration response data are expressed as mean ± SEM.

In stably-transfected CHO-A_1_R cells, VCP746, neladenoson, and capadenoson were all partial agonists for calcium mobilization relative to the prototypical full agonist NECA (Figure 1A), yet displayed full agonism for inhibition of cAMP accumulation (Figure 1B). The calcium response was sensitive to pre-treatment with pertussis toxin (*data not shown*), suggesting that, like cAMP inhibition and ERK1/2 phosphorylation (Germack and Dickenson, 2004), it is downstream of canonical Gα_i/o_-coupling. Analysis of bias, using NECA as a reference agonist and cAMP inhibition as the reference pathway, demonstrated that the synthetic ligands were biased *away* from calcium mobilization relative to cAMP (Figure 1C), a property that is reported to be predictive of a lower propensity for bradycardia.

However, neladenoson and capadenoson differed from the VCP746 profile for cell survival (1D), and phosphorylation of ERK1/2 (Figure 1E) and Akt1/2/3 (Figure 1F; Supplementary Table S1), being biased away from these endpoints compared with NECA and VCP746 (Figure 1C; Supplementary Figure S1). Interestingly, despite the structural similarity of the two investigational agents, neladenoson consistently displayed lower potency than capadenoson across all assays, particularly for protein phosphorylation (Figures 1E,F). Neladenoson, like VCP746, is biased away from Ca^2+^ influx relative to the cAMP pathway, a profile linked to reduced adenosine-like side effects. However, unlike VCP746, neladenoson shows additional bias away from the MAPK pathway.

### Functional Adenosine Receptor Subtype Selectivity and Differential Biased Agonism at the Adenosine A_2B_ Receptor

**FIGURE 2:**
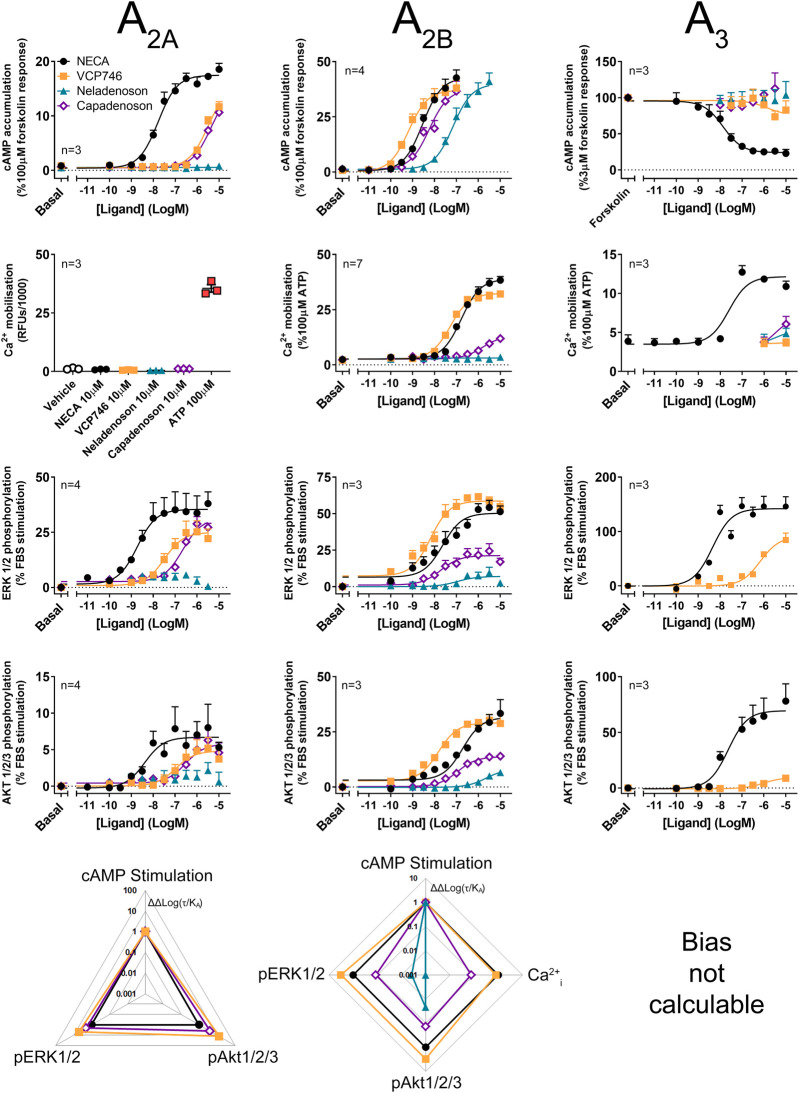
Multi-pathway profiling at the A_2A_R, A_2B_R and A_3_R. Adenosine receptor agonism was analyzed in CHO-hA_2A_R (left panels), CHO-hA_2B_R **(center panels)** and CHO-hA_3_R cells **(right panels)**. Cells were analyzed in cAMP **(top row)**, calcium **(second row)**, and phosphorylation of ERK1/2 **(third row)** and Akt1/2/3 **(fourth row)**, with calculated bias shown in the lower row. As bias data are normalized to NECA and the cAMP pathway, where there is no response to NECA at a pathway, ligand-induced cAMP in a cell line, bias cannot be calculated. Statistical analyses of bias are shown in Supplementary Figure S1. Concentration response data are expressed as mean ± SEM.

As all adenosine receptor subtypes potentially contribute to the *in vivo* activity of AR agonists, the ligands were evaluated in CHO cells stably expressing the A_2A_R, A_2B_R or A_3_R subtypes. Compared with the reference agonist NECA, VCP746 had high potency at the A_2B_R and weak activity at the A_2A_R and little or no activity at the A_3_R (Figure 2; Supplementary Figure S2; Supplementary Tables S2–S4). Capadenoson stimulated other AR subtypes with an order of selectivity of A_1_ > A_2B_ > A_2A_ >> A_3_. In contrast, neladenoson had no measurable activity at the A_2A_R or A_3_R, but activated the A_2B_R as a partial, biased agonist except for cAMP inhibition where it was a low potency full agonist (Figure 2; Supplementary Tables S2–S4).

The signaling profile of neladenoson, capadenoson, and VCP746 at the A_2B_R revealed marked differences (Figure 2). In contrast to the A_1_R, VCP746 was a non-biased full agonist at the A_2B_R, with NECA-like activity for inhibition of cAMP accumulation, calcium mobilization, ERK1/2, and Akt1/2/3 phosphorylation. On the other hand, despite being a full agonist for cAMP accumulation at the A_2B_R, capadenoson was a weak, partial agonist, biased away from calcium mobilization, ERK1/2, and Akt1/2/3 phosphorylation relative to NECA. As with the A_1_R, neladenoson was less potent than capadenoson at the A_2B_R subtype, showing bias away from ERK1/2 and Akt1/2/3 phosphorylation relative to NECA, and had no detectable activity on calcium mobilization (Figure 2; Supplementary Table S3; Supplementary Figure S1).

Agonist activity at the A_2A_R was also different for neladenoson. In contrast to capadenoson and VCP746, which were weak, partial, non-biased agonists, neladenoson did not activate the A_2A_R subtype (Figure 2; Supplementary Table S2). Of the test agents, only VCP746 showed agonist activity at the A_3_R (in pERK1/2; Figure 2), and was a low affinity antagonist for A_3_R-stimulated calcium mobilization and pERK1/2 (Supplementary Figure S2). Taken together, the pharmacological characterization of these compounds at adenosine receptor subtypes shows that neladenoson is a selective A_1_R biased agonist, with biased, weak agonism at the A_2B_R subtype, while VCP746 is a biased A_1_R agonist and potent unbiased agonist at A_2B_R.

**FIGURE 3:**
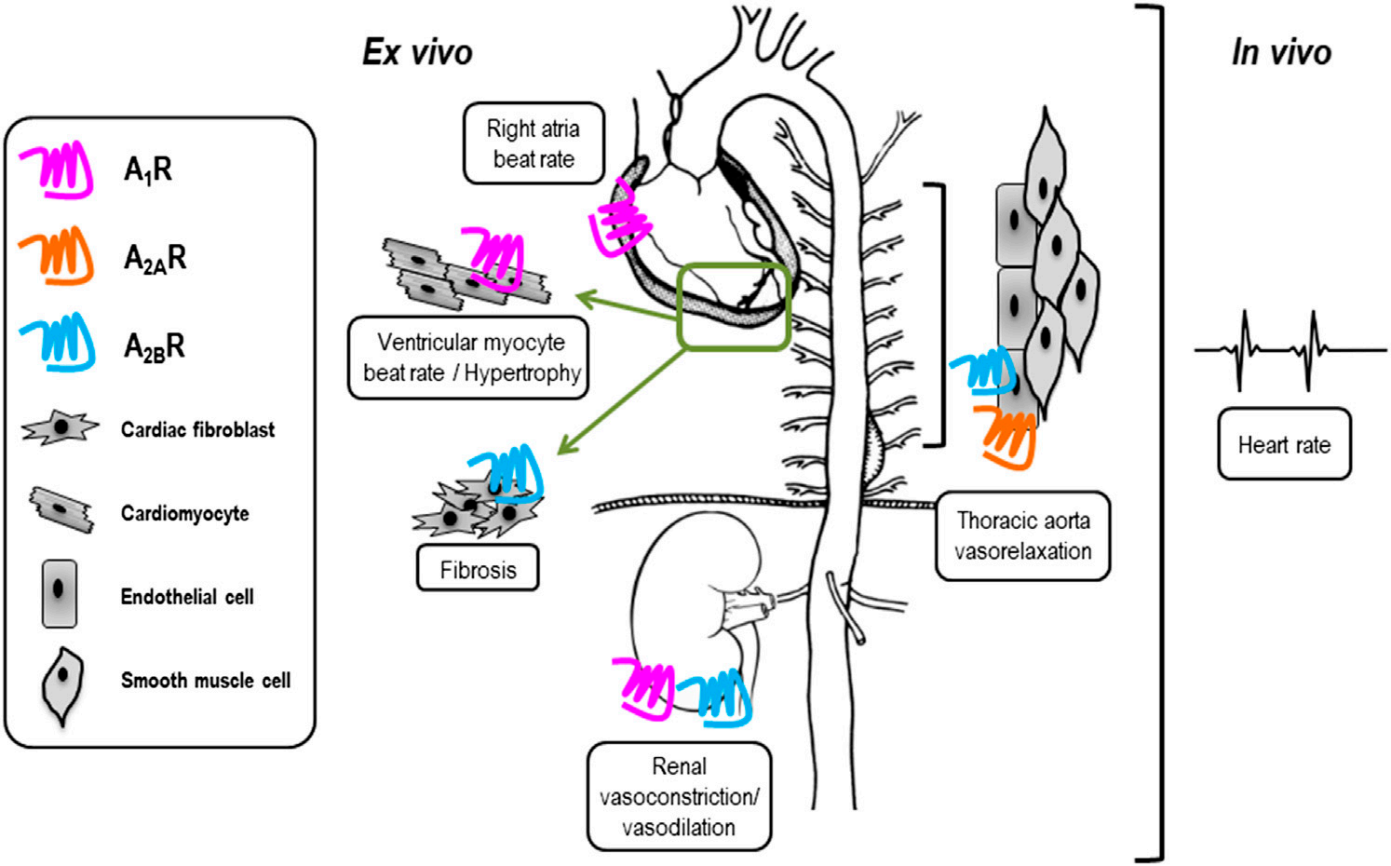
Tissue-specific expression of adenosine receptors may influence side-effect profile of compounds used *in vivo*. VCP746 and neladenoson were studied further in the indicated *ex vivo* and *in vivo* assays (boxed) to determine expected side-effect profiles. Known sites of primary functional adenosine receptor expression are indicated by blue (A_1_R), orange (A_2A_R) and pink (A_2B_R) receptors.

In order to broadly assess the physiological and pathophysiological effects of VCP746 and neladenoson in heart failure, these compounds were examined in a range of *ex vivo* and *in vivo* models to study beat/heart rate, hypertrophy/remodeling, aortic vasorelaxation, and renal vasoconstriction and vasorelaxation (Figure 3).

### VCP746 and Neladenoson Are Anti-hypertrophic in Cardiomyocytes

**FIGURE 4:**
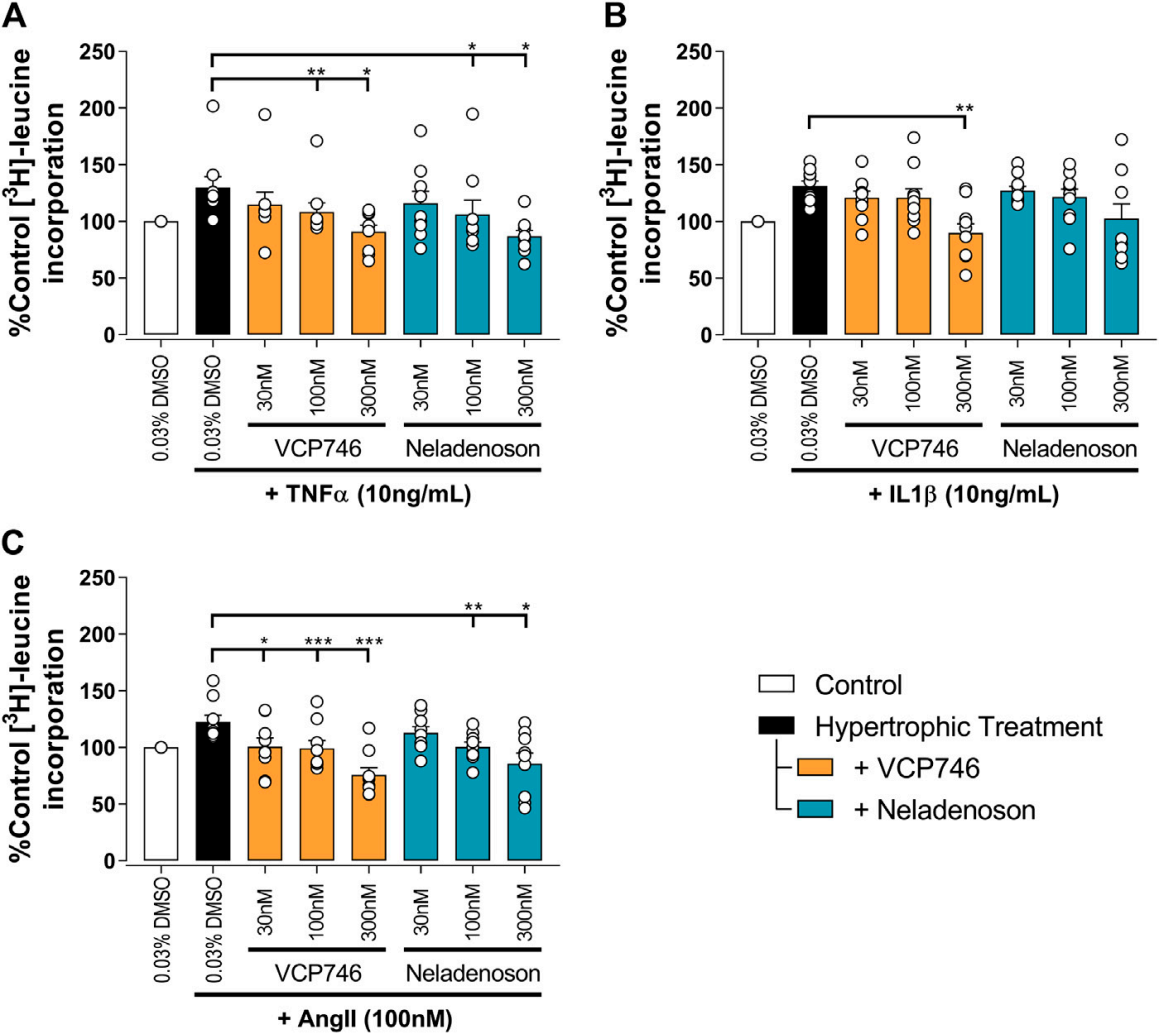
VCP746 and neladenoson reduce cardiomyocyte hypertrophy. Rat primary neonatal ventricular cardiomyocytes were exposed to hypertrophic stimuli TNFα (10 ng/ml; A; n = 9), IL1β (10 ng/ml; B; n = 10) or AngII (100 nM; C; n = 9) for 72 h after 2 h pretreatment with the indicated adenosine receptor agonists. Hypertrophy was assessed by incorporation of ^3^H-leucine as an indicator of protein synthesis. Statistical significance was assessed by repeated measures one-way ANOVA with Dunnett’s multiple comparisons, compared with hypertrophic treatment (black bar) alone (*p < 0.05, **p < 0.01, ***p < 0.001).

Putative anti-remodelling effects were assessed by [^3^H]-leucine incorporation in cardiac myocytes as a marker of hypertrophy and remodeling associated with chronic heart failure. The anti-hypertrophic effect of adenosinergic agonists, including VCP746, is believed to be mediated by A_1_R (Liao et al., 2011; Chuo et al., 2016). In the current study TNFα, IL1β, or Ang II induced neonatal ventricular cardiomyocyte (NVCM) hypertrophy that was prevented by pre-treatment with VCP746 or neladenoson in a concentration-dependent manner (Figure 4). These effects occurred over the same range of concentrations for both drugs, despite the 10-fold higher potency of neladenoson for A_1_R mediated cAMP inhibition (Figure 1). None of the compounds showed a significant effect on NVCM viability as measured by both PI staining or LDH release assays (Supplementary Figure S3), indicating that the inhibitory effects are not due to a reduction on cell viability.

### Both VCP746 and Neladenoson Have Anti-fibrotic Effects in Cardiac Fibroblasts

**FIGURE 5:**
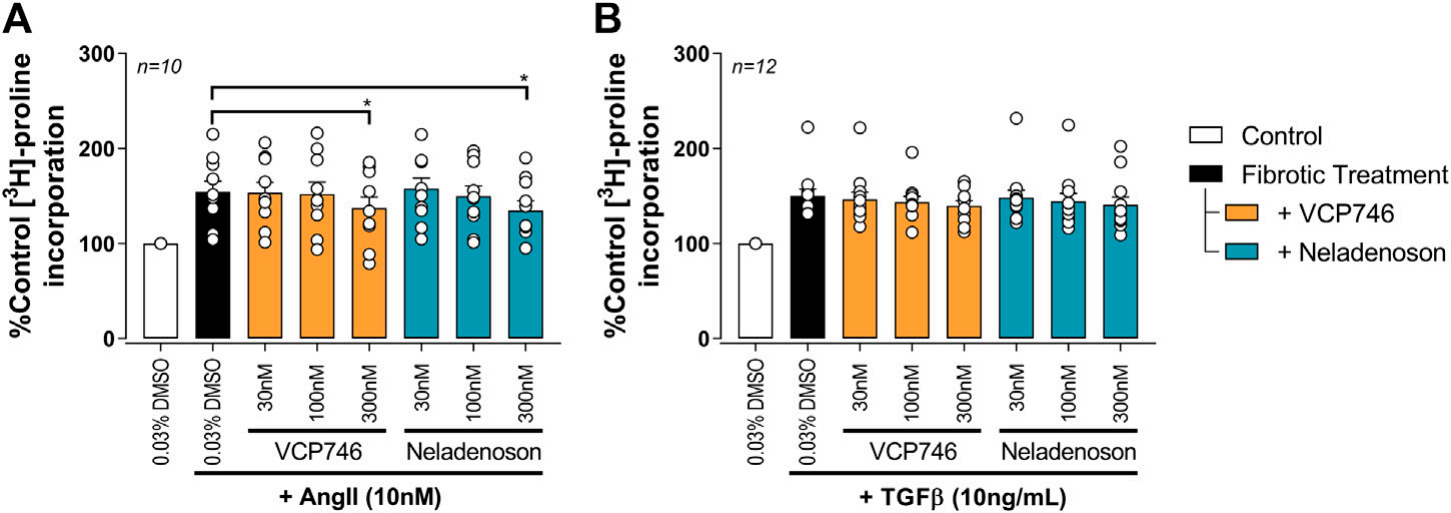
Adenosinergic agonists show some anti-fibrotic activity in rat primary neonatal cardiac fibroblasts. Fibrosis was measured by the incorporation of ^3^H-proline as an indicator of collagen formation. After 72 h 10 nM AngII **(A)** or 10 ng/ml TGFβ **(B)** treatment, VCP746 and neladenoson pre-treatment (2 h) inhibited the AngII-fibrotic effect only, at 300 nM (repeated measures one-way ANOVA, Dunnett’s multiple comparisons, in comparison to fibrotic treatment alone; *(*p* < 0.05)).

In cardiac fibroblasts, Ang II and tumor growth factor-beta (TGFβ) significantly increased [^3^H]-proline incorporation, a marker of fibrosis (Figure 5). Pre-treatment with either VCP746 or neladenoson (300 nM) reduced the effect of Ang II by 40% (Figure 5A), putatively by activation of adenosine A_2B_ receptors (Vecchio et al., 2016a), although neither agent affected [^3^H]-proline incorporation driven by TGFβ (Figure 5B). These effects are consistent with the signaling profile of the agonists at the A_2B_R since both are able to activate this receptor.

### VCP746 and Neladenoson Display Limited and Differential Effects on Beat/Heart Rate *In Vitro* and *In Vivo*

One of the major barriers to the use of adenosine A_1_R agonists in the clinic is A_1_R-mediated negative chronotropy i.e. bradycardia. To evaluate the relative propensity to modulate beat rate, we compared VCP746 and neladenoson head-to-head in primary NVCMs, rat atria *ex vivo*, and in conscious rats by telemetry.

**FIGURE 6:**
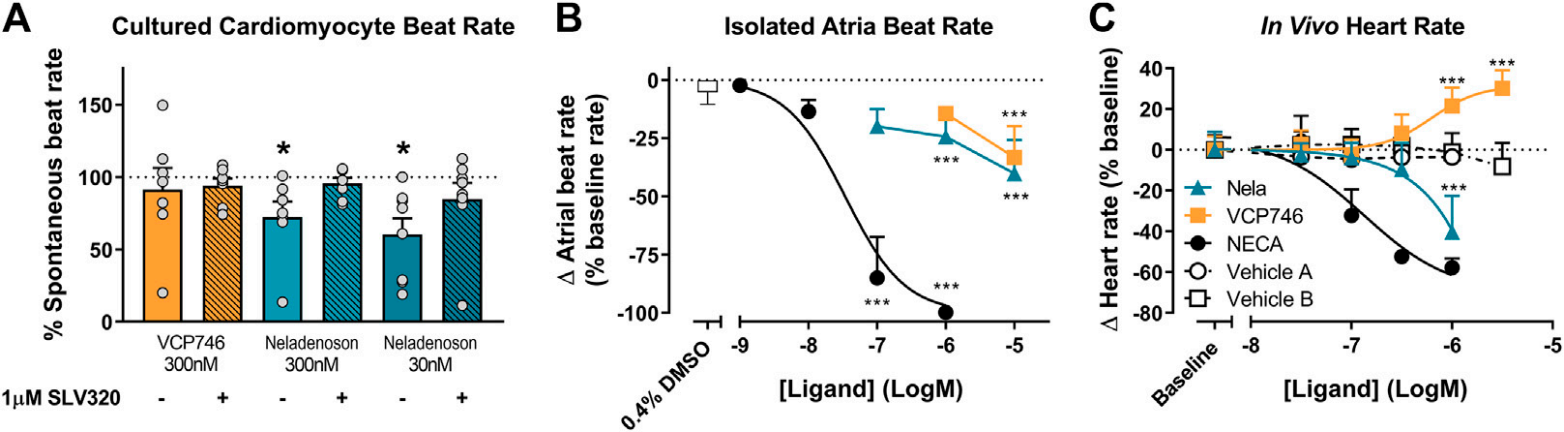
*Ex vivo* and *in vivo* A_1_R-mediated chronotropic effects. In isolated neonatal ventricular cardiomyocytes, VCP746 (n = 7) had no effect on the spontaneous contraction rate of cells **(A)**, while neladenoson (30 nM: n = 7; 300 nM: n = 8) showed a significant depressive effect, which is completely abrogated by pre-treatment with the A_1_R antagonist SLV320 (statistics were performed on raw data; paired Student’s t-test, comparison of VCP746/neladenoson-treated cells to the same cells prior to treatment; *(*p* < 0.05)). Data are mean ± SEM. In isolated atria **(B)**, NECA produced a concentration-dependent effect (n = 8), while only minor effects of VCP746/neladenoson (n = 4–9) were observed at high concentrations (1 or 10 μM; one-way ANOVA compared with vehicle alone; ***(*p* < 0.01)). **(C)** Adenosinergic compounds were also tested *in vivo*. NECA (n = 6) decreased heart rate relative to vehicle A (n = 2). Neladenoson (n = 4) significantly reduced heart rate at 1µM relative to vehicle B (n = 7), while VCP746 (n = 5) increased heart rate at high concentrations *in vivo* (two-way ANOVA, Sidak’s multiple comparisons, compared with vehicle **B)**. Data for **(B)** and **(C)** are expressed as mean ± SD.

In primary rat NVCMs, neladenoson (30 and 300 nM) produced a small, but significant reduction in spontaneous beat rate (Figure 6A), whereas VCP746 (300 nM) was without significant effect. The reduction of beat rate after treatment with neladenoson was reversed by pre-treatment with an A_1_R subtype-selective antagonist, SLV320, indicating that this effect is A_1_R-mediated (Figure 6A).

In rat isolated right atria, neladenoson and VCP746 had a minimal effect on the beat rate in contrast to NECA, which decreased beat rate in a concentration-dependent manner (pIC_50_ = 7.5 ± 0.3, n = 8; Figure 6B). By using AR subtype-specific agonists and the A_1_R specific antagonist SLV320 we confirmed that the bradycardic response was also almost entirely A_1_R-mediated. The A_1_R selective agonist 2-Me-CCPA maximally inhibited beat rate in a manner similar to the non-selective agonist, NECA, whereas CGS21680 (A_2A_R selective) or BAY60-6583 (A_2B_R selective) had no significant effect in the same concentration range (Supplementary Figure S4A). Additionally, pre-treatment with the selective A_1_R antagonist SLV320, abrogated the chronotropic effect of NECA, further confirming the involvement of A_1_R (Supplementary Figure S4B).

Likewise, in conscious rats, increasing plasma concentrations of NECA reduced telemetered heart rate in a concentration-dependent manner (pIC_50_ = 6.9 ± 0.2, n = 6; Figure 6C). Neladenoson (1 μM) produced a decrease in heart rate, whereas VCP746 (1–3 μM) produced a modest, concentration-dependent increase in heart rate (Figure 6C).

Collectively the data show that AR-mediated chronotropic effects are A_1_R-dependent and suggest that, unlike prototypical AR agonists, biased agonists such as VCP746 and neladenoson have limited effects on cardiomyocyte or isolated atria beat rate *ex vivo,* and heart rate in rodents *in vivo*, further strengthening the link between the bias profile of both of these compounds and the lack of chronotropic effects, thus providing a potential route for avoidance of on-target mediated side effects.

### VCP746 and Neladenoson Induce Renal Vasodilation by A_2B_R Agonist Activity

Patients with heart failure exhibit abnormal cardio-renal hemodynamics that ultimately exacerbates the disease. Adenosine receptors play an important role in the hemodynamic balance in the kidney; adenosine induces A_1_R-mediated cortical vasoconstriction and A_2A_R/A_2B_R-mediated medullar vasodilation, reducing filtration fraction in order to recover from negative energy balance in the kidney. Accordingly, effects in the kidney may potentially have implications for any adenosine receptor agonist synthesized for clinical use.

**FIGURE 7:**
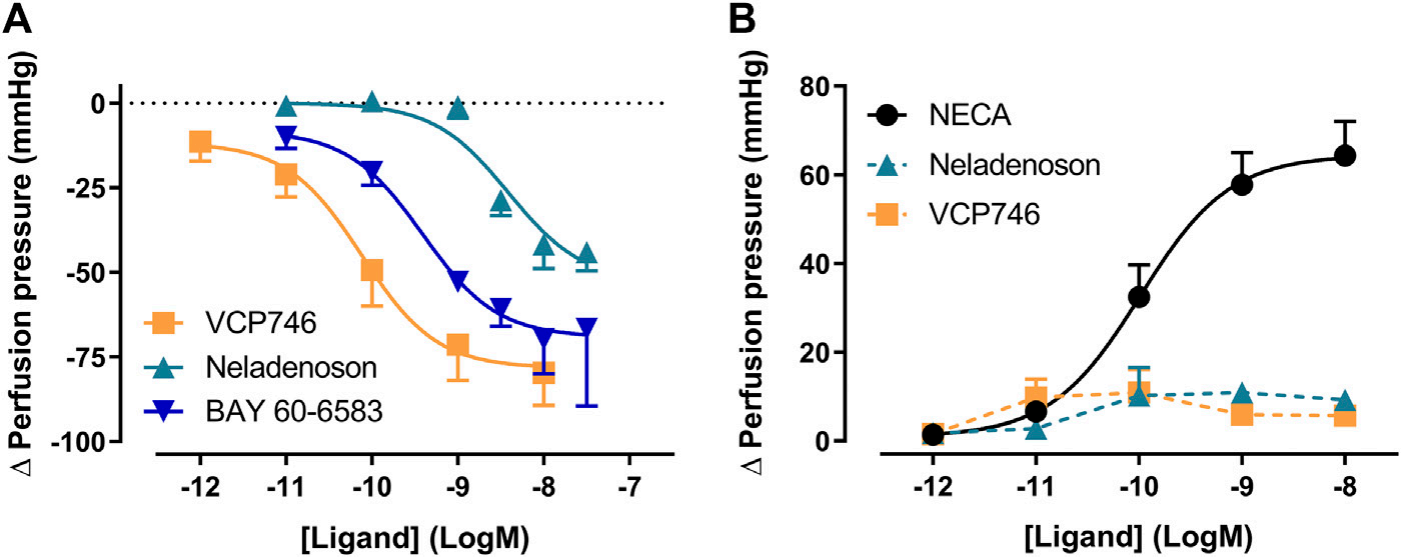
VCP746 and neladenoson induce A_2B_R-mediated renal vasodilation but no A_1_R-mediated vasoconstriction. **(A)** Renal vasodilation was measured by administering VCP746 or the A_2A_R/A_2B_R agonist BAY 60-6583 to methoxamine-stimulated rat kidney and measuring resulting perfusion pressure (n = 3). The concentration-dependent vasodilation produced, with order of potency VCP467 > BAY 60-6583 > neladenson suggest an A_2B_R-mediated mechanism of action, which was confirmed using selective antagonists (Supplementary Figure S5). **(B)** In order to measure renal vasoconstriction, kidneys were stimulated with methoxamine/forskolin and then NECA (n = 6), neladenoson (n = 6) or VCP746 (n = 5) was applied. NECA induced a potent constrictor activity, with no response observed to neladenoson/VCP746. This was confirmed to be an A_1_R-mediated effect (Supplementary Figure S5). Data are expressed as mean ± SEM; where error bars are not visible, they are contained within the dimensions of the symbol.

Using the α_1_-adrenoceptor agonist methoxamine to elevate vascular tone, we investigated renal vasodilation. The adenosine A_2B_R agonist BAY 60-6583, VCP746, and neladenoson all induced vasodilation, with a potency (pEC_50_) of order VCP746 (10.1 ± 0.3, n = 3) > BAY 60-6583 (9.4 ± 0.4, n = 3) > neladenoson (8.4 ± 0.2, n = 3; Figure 7A). Since the BAY 60-6583 response is sensitive to MRS1754, but not SCH442426 (Supplementary Figure S5A), it suggests that the response is A_2B_R-, rather than A_2A_R-mediated. This would be consistent with VCP746 showing greater potency and efficacy at the A_2B_R compared with neladenoson (Figures 2, 7A).

In order to study effects on vasoconstriction, methoxamine-treated kidneys were treated with forskolin to activate adenylate cyclase and reduce vascular tone. Under these conditions, NECA induced renal vasoconstriction (Figure 7B) in a concentration dependent-manner (pEC_50_ = 10.0 ± 0.2, n = 6). Similarly, the A_1_R-selective agonist, 2-Me-CCPA, induced a vasoconstrictor response that was abolished by the A_1_R-specific antagonist, SLV320 (Supplementary Figure S5B) indicating that the renal vasodilation is A_1_R-dependent. However, VCP746 and neladenoson had no effect on the vasoconstrictor response (Figure 7B). Since the response is sensitive to A_1_R activation, the lack of effect after VCP746 or neladenoson suggests that the bias profile of these compounds contributes to their lack of renal vasoconstrictor activity.

### Neither VCP746 nor Neladenoson Induce Endothelium-dependent Thoracic Aorta Relaxation

**FIGURE 8:**
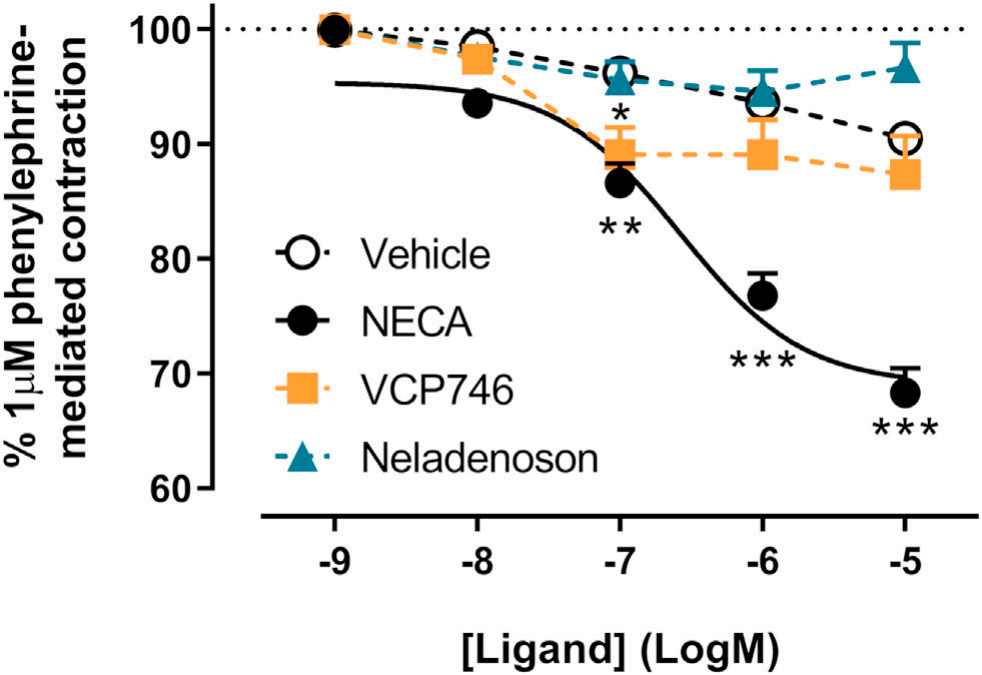
NECA, but not VCP746 or neladenoson induces endothelium-dependent relaxation of rat thoracic aorta. NECA concentration-dependently inhibited phenylephrine-mediated contraction (pEC_50_ 6.6 ± 0.2) while VCP746 and neladenoson displayed negligible effects (n = 3). Antagonist studies suggest this is a predominately A_2A_R-mediated mechanism (Supplementary Figure S6). Data are expressed as mean ± SEM; where error bars are not visible, they are contained within the dimensions of the symbol. Statistical significance treatment was assessed by repeated measures mixed effects model with Dunnett’s multiple comparisons, compared with vehicle (*p < 0.05, **p < 0.01, ***p < 0.001).

In order to confirm subtype selectivity, VCP746 and neladenoson were tested for effects on rat thoracic aorta relaxation, in the presence and absence of endothelium. NECA relaxed the aorta in an endothelium-dependent A_2A_R-mediated manner (Figure 8; Supplementary Figure S6). In contrast, neither VCP746 nor neladenoson had any effect on the aorta (Figure 8), broadly consistent with their relatively low potency (VCP746) or lack of activity (neladenoson) at the A_2A_R (Figure 2).

## Discussion

The A_1_R has been a focus of heart failure drug discovery efforts for over 2 decades. The cardioprotective effects of adenosine are well described, but efforts to develop selective A_1_R agonists have been limited by on-target mediated bradycardia, atrioventricular block, and other extra-cardiac undesired effects. Several approaches have been taken to the design of novel agents to mitigate these risks. Bayer developed the A_1_R partial agonists capadenoson and neladenoson (a pro-drug of an active and more soluble metabolite; Meibom et al., 2017), whereas other pre-clinical work identified biased A_1_R agonists that exhibit specific signaling profiles focused on avoiding on-target mediated adverse effects. The A_1_R subtype is a Gα_i/o_-coupled receptor that couples pleiotropically to multiple downstream endpoints, including inhibition of cAMP accumulation, MAPK activation, and increased intracellular calcium mobilization. Thus, linking a signaling pathway (or subset of pathways) to a specific physiological outcome represents a mechanism whereby agents can be developed that avoid on-target side effects on the kidney, peripheral blood vessels, or the heart.

VCP746 is a tool biased A_1_R agonist that preferentially signals away from intracellular calcium mobilization compared with inhibition of cAMP accumulation (Baltos et al., 2016). Growing evidence points to agonist-induced increases in calcium flux as a predictor of A_1_R-mediated side effects (Valant et al., 2014; Baltos et al., 2016; Greene et al., 2016). We have shown that the A_1_R bias signaling profile of capadenoson, neladenoson and VCP746 are broadly similar in that they are biased away from stimulation of intracellular calcium mobilization. This phenotype translated to primary cardiomyocytes, isolated rat atria, and *in vivo* in telemetered rats, where both neladenoson and VCP746 had little effect on beat/heart rate, unlike the prototypical adenosine analogue, NECA. Interestingly, at equi-effective concentrations for A_1_R-mediated inhibition of cAMP signaling, neladenoson inhibited the beat rate of ventricular cardiomyocytes and heart rate *in vivo* in contrast to VCP746, suggesting alternative signaling pathways are involved in this process.

Nonetheless, in the clinic, capadenoson and neladenoson had little effect on heart rate in trials in patients with angina pectoris, atrial fibrillation, or congestive heart failure (Shah et al., 2019; Voors et al., 2019; Clinical Trial NLM Identifiers: NCT00568945, NCT03098979, NCT02992288) suggesting that bias in the cAMP-calcium signaling balance downstream of the A_1_R is a critical predictor for avoiding bradycardia (Zablocki et al., 2004; Albrecht-Küpper et al., 2012).

A further consideration for A_1_R agonists is their potential for deleterious effects on renal hemodynamics and a number of groups have developed A_1_R *antagonists* for the treatment of impaired renal function in congestive heart failure (Vallon et al., 2008). This approach was predicated on stimulating renal vessel relaxation and inhibiting the tubuloglomerular feedback mechanism, thus increasing urine output without worsening glomerular filtration rate. Such a mechanism suggests that A_1_R *agonists* might impair renal function. However, our studies show that VCP746 and neladenoson lack renal vasoconstrictor effects in the methoxamine/forskolin-treated kidney. Again, this indicates that the signaling profile at the A_1_R and/or ancillary pharmacology of these two agents is beneficial when compared with non-selective/non-biased adenosine receptor agonists that cause vasoconstriction. In fact, both neladenoson and VCP746 caused renal vasorelaxation, mediated by the adenosine A_2B_ receptor, which explains the 30-fold discrepancy in potency between the two agents in favor of VCP746. In turn this offers a potential explanation for the small, concentration-dependent increase in heart rate observed with VCP746 *in vivo* that may well be a compensatory response to the increased renal vasorelaxation whilst the rats remain normotensive.

Clinically, neladenoson either had no (Shah et al., 2019) or marginal (Voors et al., 2019) effects on heart rate, a profile largely predicted by pre-clinical models. However, the compound failed to improve cardiac and non-cardiac abnormalities in phase II trials in heart failure patients with either preserved or reduced ejection fraction, and slightly worsened renal function in the latter population (Shah et al., 2019; Voors et al., 2019). This clearly differs from the pre-clinical profile, where neladenoson was cardioprotective in a left anterior descending artery occlusion-induced ischemia model in rats (Meibom et al., 2017).

The failure of neladenoson in these clinical trials poses a number of questions for adenosine receptor-targeting heart failure therapeutics. Despite the clinically-proven cardioprotective effects of adenosine (McIntosh and Lasley, 2012; Headrick et al., 2013; Randhawa and Jaggi, 2016), it might be that selective A_1_R activation, differential A_1_R bias, or targeting patients with chronic, rather than acute, heart failure, could be responsible for the lack of efficacy of neladenoson. Whilst neladenoson has the critical cAMP-calcium bias required for improving therapeutic index, it is also highly biased away from the MAPK pathways, ERK1/2 and Akt1/2/3 phosphorylation. This may be undesirable since ERK1/2 phosphorylation plays a role in cardioprotection (Germack and Dickenson, 2005; Reid et al., 2005; Kovacs et al., 2009; Rose et al., 2010), while Akt1/2/3 phosphorylation is generally considered a pro-survival pathway utilized by many cardioprotective agents (Matsui and Rosenzweig, 2005; Solenkova et al., 2006; Manning and Cantley, 2007; Kovacs et al., 2009). It remains to be seen whether therapeutic agents can be developed that have A_1_R cAMP-calcium bias, without the bias away from MAPK signaling. This signaling profile may explain the greater potency of VCP746 in reducing humoral- and inflammation-induced cardiomyocyte hypertrophy compared with neladenoson – herein and (Chuo et al., 2016) – despite neladenoson being *more* potent than VCP746 for cAMP inhibition.

With respect to selectivity, our recombinant cell signaling data showed that neladenoson activated neither the A_2A_R or A_3_R subtypes, and (unlike VCP746) is a biased agonist at the A_2B_R (away from MAPK while calcium signaling was undetectable). Activation of A_2B_R reduces fibrosis in heart failure (Dubey et al., 1997, 1998, 2001; Chen et al., 2004), though the signaling pathway(s) responsible for this are not well defined. Here we showed equivalence for neladenoson and VCP746 for anti-fibrotic efficacy in cardiac fibroblasts, and previous studies have shown that VCP746 exhibits potent A_2B_R-dependent anti-fibrotic activity (Vecchio et al., 2016a). Thus, appropriate A_2B_R signaling profiles for adenosine receptor agonists may be required in heart failure therapeutics. These issues of selectivity and bias are important in the context of treating chronic, as opposed to acute, heart failure, where anti-hypertrophic and anti-fibrotic activity would be desirable. Additionally, the lack of clinical translation calls into question the predictive capacity of rodent models of heart failure (Houser et al., 2012) or their translation to specific stages of the disease in patients.

The concept of biased agonism faces a number of challenges in linking signaling profiles to clinical endpoints and improved therapeutic indices. Despite promising preclinical data, biased agonists have yet to fulfill their full promise in the clinic: the β-arrestin-biased angiotensin AT1 receptor agonist, TRV 027, failed to meet its primary endpoint in a phase IIb study of patients with acute heart failure (Pang et al., 2017). In addition to improving the pre-clinical assessment of what constitutes a biased agonist (e.g. for µ-opioid receptors (Conibear and Kelly, 2019)), it is critical to understand the pharmacology of agents that have been developed and clinically-evaluated without reference to the phenomenon of signal bias. In this study we comprehensively compared *in vitro*, *ex vivo*, and *in vivo* properties of the investigational A_1_R agonist, neladenoson, compared to VCP746, a tool A_1_R biased agonist. Retrospective analysis of the signaling profile of neladenoson reinforces the cellular predictor (cAMP-calcium signaling bias) for its lack of effect on heart rate in pre-clinical models and patients. Although the reasons for the lack of clinical efficacy remain unknown, it is possible that fine tuning activity in other pathways (e.g. MAPK) and/or adenosine receptors (e.g. A_2B_R) might represent valid future approaches for the development of novel agents.

## Data Availability

The raw data supporting the conclusions of this article will be made available by the authors, without undue reservation.
