# Supplementary material for: Pharmacological Insights Into Safety and Efficacy Determinants for the Development of Adenosine Receptor Biased Agonists in the Treatment of Heart Failure
Source: Front Pharmacol. 2021 Mar 11;12:628060. doi: 10.3389/fphar.2021.628060 (PMC7991592; doi:10.3389/fphar.2021.628060)
Supplement: Supplementary file 1 [file datasheet1.docx]

**SUPPLEMENTARY TABLES**

| **Pathway/**  **Ligand** | **cAMP inhibition** (6) | | **Ca^2+^_I_** (4) | | **pERK1/2** (5) | | **pAKT1/2/3** (3) | | **Cell Survival** (3) | |
| --- | --- | --- | --- | --- | --- | --- | --- | --- | --- | --- |
|  | **pEC_50_** | **E_max_**  **(%Fsk)** | **pEC_50_** | **E_max_**  **(%ATP)** | **pEC_50_** | **E_max_**  **(%FBS)** | **pEC_50_** | **E_max_**  **(%FBS)** | **pEC_50_** | **Span (%Basal)** |
| **NECA** | 8.8 ± 0.1 | 13.5 ± 2.2 | 7.6 ± 0.1 | 38.0 ± 1.2 | 9.1 ± 0.1 | 87 ± 4.1 | 8.5 ± 0.1 | 109 ± 4 | 9.2 ± 0.2 | 21.6 ± 1.8 |
| **VCP746** | 7.7 ± 0.1 | 13.4 ± 3.2 | 6.8 ± 0.5 | 8.1 ± 1.3 | 8.2 ± 0.1 | 105 ± 3 | 7.7 ± 0.1 | 91 ± 5 | 8.3 ± 0.3 | 21.1 ± 3.1 |
| **Capadenoson** | 9.5 ± 0.1 | 16.5 ± 2.1 | 7.6 ± 0.1 | 19.2 ± 0.9 | 8.9 ± 0.1 | 102 ± 4 | 8.5 ± 0.1 | 93 ± 3 | 9.5 ± 0.2 | 22.5 ± 1.7 |
| **Neladenoson** | 8.9 ± 0.1 | 13.2 ± 2.7 | 7.2 ± 0.2 | 16.6 ± 1.1 | 7.1 ± 0.1 | 117 ± 4 | 6.6 ± 0.1 | 80 ± 5 | 9.0 ± 0.2 | 23.9 ± 1.9 |

**Supp Table 1.** Potencies (pEC_50_) and E_max_/Span of responses at A_1_R (relative to indicated control) of the adenosinergic compounds from data in Figure 1. Data are mean ± SEM and experimental *n* are indicated in brackets.

| **Pathway/**  **Ligand** | **cAMP stimulation** (3) | | **Ca^2+^_I_** (3) | | **pERK1/2** (3) | | **pAKT1/2/3** (4) | |
| --- | --- | --- | --- | --- | --- | --- | --- | --- |
|  | **pEC_50_** | **E_max_**  **(%Fsk)** | **pEC_50_** | **E_max_**  **(%ATP)** | **pEC_50_** | **E_max_**  **(%FBS)** | **pEC_50_** | **E_max_**  **(%FBS)** |
| **NECA** | 7.8 ± 0.1 | 17.5 ± .5 | ---- | ---- | 8.7 ± 0.2 | 35.4 ± 2 | 8.4 ± 0.3 | 6.7 ± .7 |
| **VCP746** | ~5.5 | ~15.6 | ---- | ---- | 7.5 ± 0.2 | 25.0 ± 2 | 7.0 ± 0.3 | 4.8 ± 0.6 |
| **Capadenoson** | ~5.4 | ~15.3 | ---- | ---- | 6.7 ± 0.1 | 31.3 ± 2 | 6.5 ± 0.3 | 5.8 ± 0.8 |
| **Neladenoson** | ---- | ---- | ---- | ----- | ---- | ---- | ---- | ---- |

**Supp Table 2.** Potencies (pEC_50_) and E_max_ responses at A_2A_R (relative to indicated control) of the adenosinergic compounds from data in Figure 2. “~” indicates true E_max_ (and therefore potency) could only be estimated, and “----” indicates no response was observed. Data are mean ± SEM and experimental *n* are indicated in brackets.

| **Pathway/**  **Ligand** | **cAMP stimulation** (4) | | **Ca^2+^_I_** (7) | | **pERK1/2** (3) | | **pAKT1/2/3** (3) | |
| --- | --- | --- | --- | --- | --- | --- | --- | --- |
|  | **pEC_50_** | **E_max_**  **(%Fsk)** | **pEC_50_** | **E_max_**  **(%ATP)** | **pEC_50_** | **E_max_**  **(%FBS)** | **pEC_50_** | **E_max_**  **(%FBS)** |
| **NECA** | 8.5 ± 0.1 | 42.7 ± 1.7 | 6.8 ± 0.1 | 38.8 ± 0.7 | 7.6 ± 0.2 | 50.3 ± 2.4 | 6.9 ± 0.1 | 28.8 ± 1.7 |
| **VCP746** | 9.2 ± 0.1 | 37.1 ± 1.4 | 7.3 ± 0.1 | 32.4 ± 0.5 | 8.2 ± 0.1 | 58.7 ± 1.8 | 7.8 ± 0.1 | 29.0 ± 0.9 |
| **Capadenoson** | 8.2 ± 0.1 | 37.3 ± 2.5 | 5.8 ± 0.2 | 13.2 ± 1.4 | 7.7 ± 0.2 | 21.3 ± 1.6 | 7.0 ± 0.1 | 13.7 ± 0.5 |
| **Neladenoson** | 7.2 ± 0.1 | 39.9 ± 1.9 | ---- | ---- | 6.8 ± 0.4 | 8.1 ± 1.4 | ~5.7 | ~8.3 |

**Supp Table 3.** Potencies (pEC_50_) and E_max_ responses at A_2B_R (relative to indicated control) of the adenosinergic compounds from data in Figure 2. “~” indicates true E_max_ (and therefore potency) could only be estimated, and “----” indicates no response was observed. Data are mean ± SEM and experimental *n* are indicated in brackets.

| **Pathway/**  **Ligand** | **cAMP stimulation** (4) | | **Ca^2+^_I_** (7) | | **pERK1/2** (3) | | **pAKT1/2/3** (3) | |
| --- | --- | --- | --- | --- | --- | --- | --- | --- |
|  | **pEC_50_** | **E_max_**  **(%Fsk)** | **pEC_50_** | **E_max_**  **(%ATP)** | **pEC_50_** | **E_max_**  **(%FBS)** | **pEC_50_** | **E_max_**  **(%FBS)** |
| **NECA** | 7.9 ± 0.1 | 24 ± 3.2 | 7.6 ± 0.2 | 12 ± 0.6 | 8.4 ± 0.1 | 147 ± 7 | 7.6 ± 0.3 | 69 ± 6 |
| **VCP746** | ~5.5 | ~76 | ---- | ---- | ~6.1 | ~91 | ~5.9 | ~9.7 |
| **Capadenoson** | ---- | ---- | ---- | ---- | ---- | ---- | ---- | ---- |
| **Neladenoson** | ---- | ---- | ---- | ---- | ---- | ---- | ---- | ---- |

**Supp Table 4.** Potencies (pEC_50_) and E_max_ responses at A_3_R (relative to indicated control) of the adenosinergic compounds from data in Figure 2. “~” indicates true E_max_ (and therefore potency) could only be estimated, and “----” indicates no response was observed. Data are mean ± SEM and experimental *n* are indicated in brackets.

**SUPPLEMENTARY FIGURES**

**
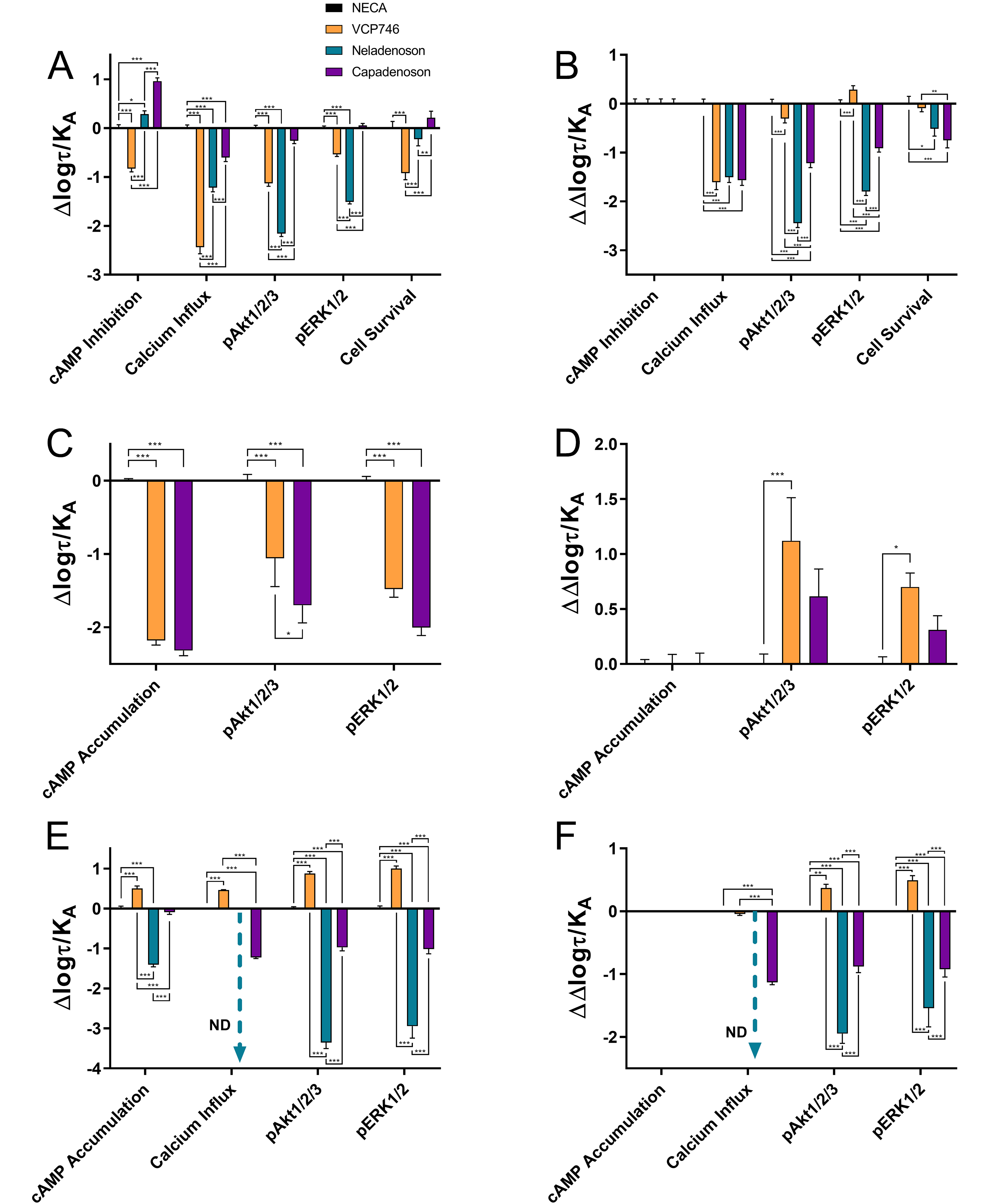
**

**Supp Figure 1**

Bias data were normalised to NECA within each cell line (**A**, **C** and **E**), before normalisation to the canonical cAMP pathway (**B**, **D** and **F**). Each experiment was performed with all ligands so that bias could be calculated within each *n*. Calcium influx bias of neladenoson at A_2B_ (E and F) was not determined (*ND*) due to the lack of calcium response. As calculated bias from each experiment contained error, data are expressed as *weighted* mean ± SEM, and therefore contain no individual data points. The weighted mean ($x̅$) is calculated by:

$$\bar{x}= \frac{\sum_{i=1}^{n} w_{i}x_{i}}{\sum_{i=1}^{n} w_{i}}$$

where $w_{i}$ is the weight of each experiment and $x_{i}$ is the mean from each experiment. The weighting of each experiment ($w_{i}$) is calculated by:

$$w_{i}= \frac{1}{\sigma_{i}^{2}}$$

where $\sigma_{i}^{2}$ is the variance (square of the standard deviation) from each experiment. The error (SEM) is propagated through by dividing $\sigma_{\bar{x}}$ (standard deviation of the weighted mean) by *n*, where:

$$\sigma_{\bar{x}}=\frac{1}{\sqrt{\sum_{i=1}^{n} w_{i}}}$$

Data for each pathway were analysed by two-way ANOVA, *(p<0.05), **(p<0.01), ***(p<0.001).


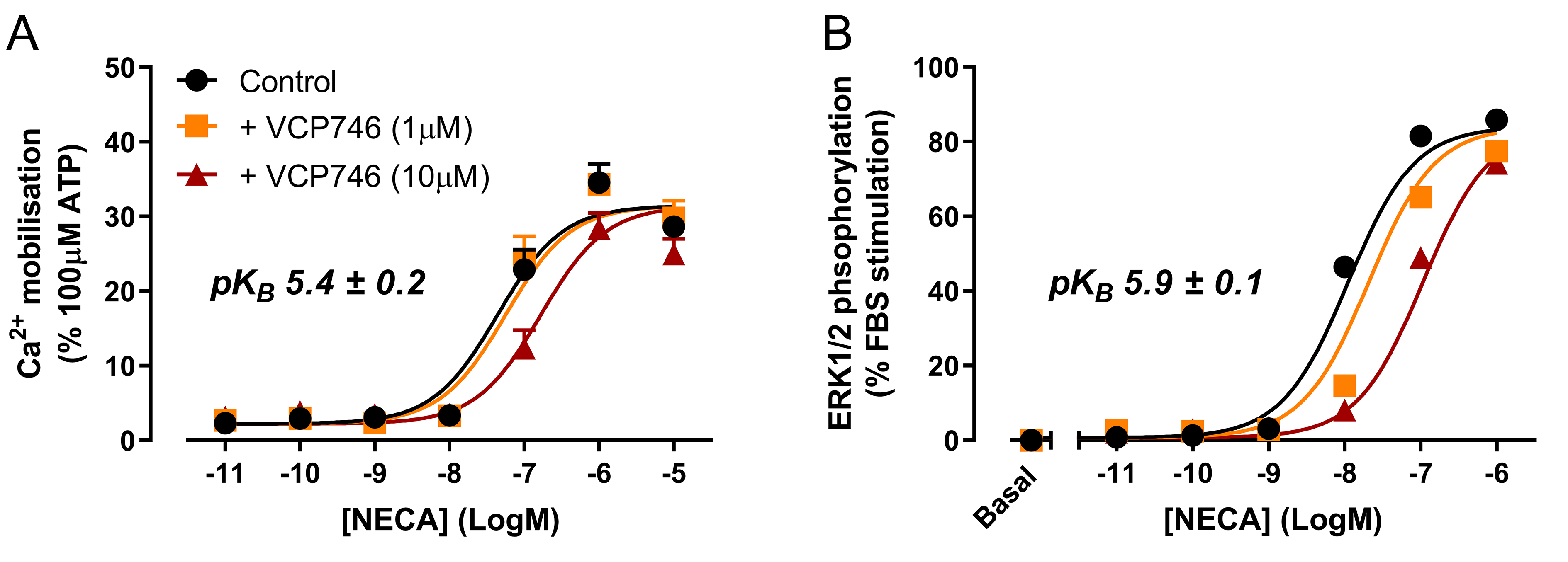


**Supp Figure 2.** VCP746 acts as a low affinity antagonist on the A_3_R. VCP746 (1μM or 10μM) was applied 15 minutes prior to NECA in calcium influx (**A**; n=4) and ERK1/2 phosphorylation assays (**B**; n=4). pK_B_ values were calculated using the Gaddum/Schild EC_50_ shift method in GraphPad Prism 8.02.


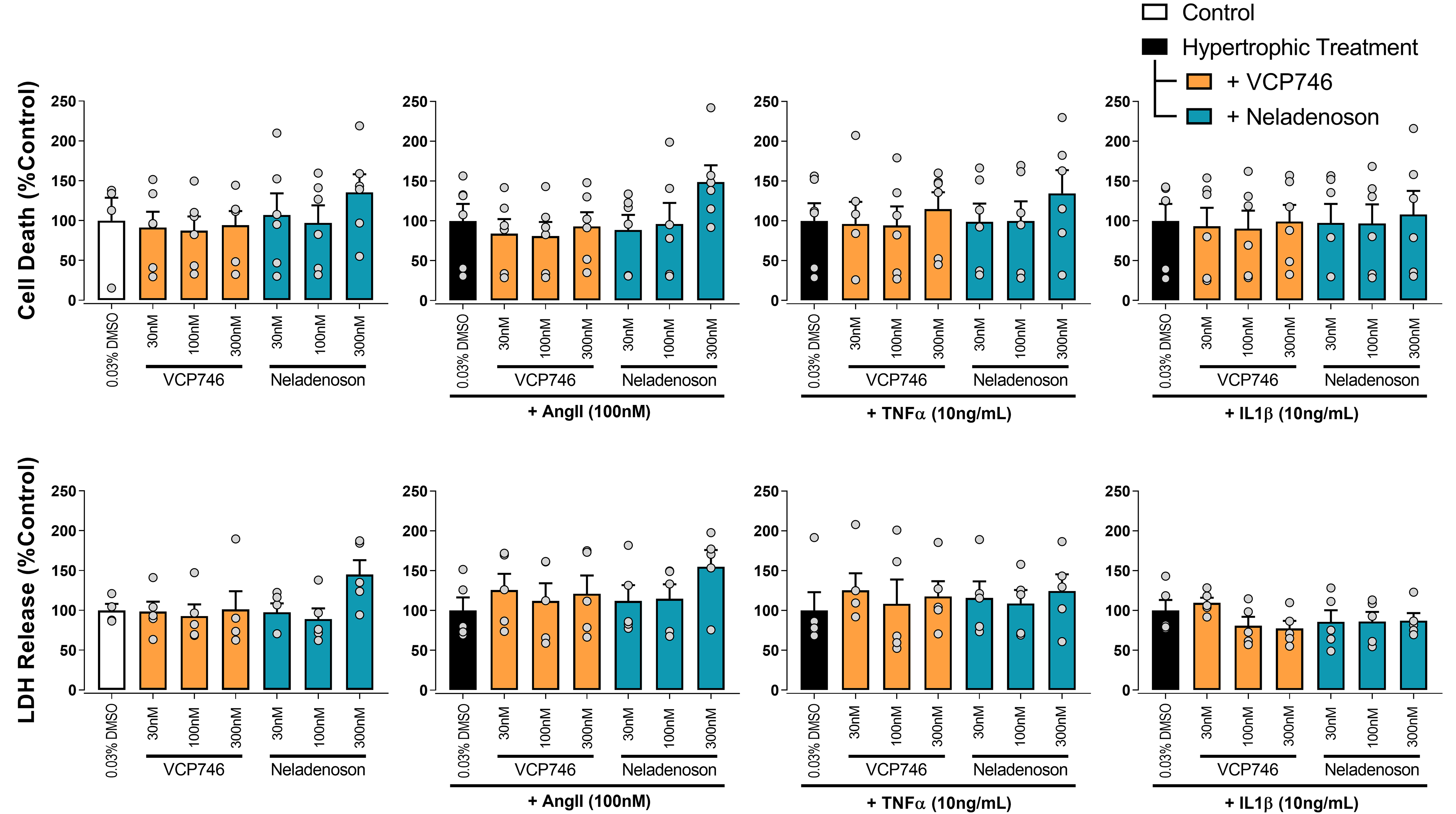


**Supp Figure 3**. Adenosinergic treatments did not affect NVCM cell viability. Rat primary neonatal ventricular cardiomyocytes treated identically to hypertrophy assays (Fig 4) were analysed for effects on viability through two methods: proportion of cells stained by PI relative to the entire population determined by Hoescht33324 (top panels; n=5); and the release of LDH from cells into the media as an indication of membrane permeability (lower panels; n=5). In both cases data are normalised to control within that hypertrophic treatment. No statistical significance was determined (repeated measures one-way ANOVA with Dunnett’s post-test, compared to control or hypertrophic treatment alone).


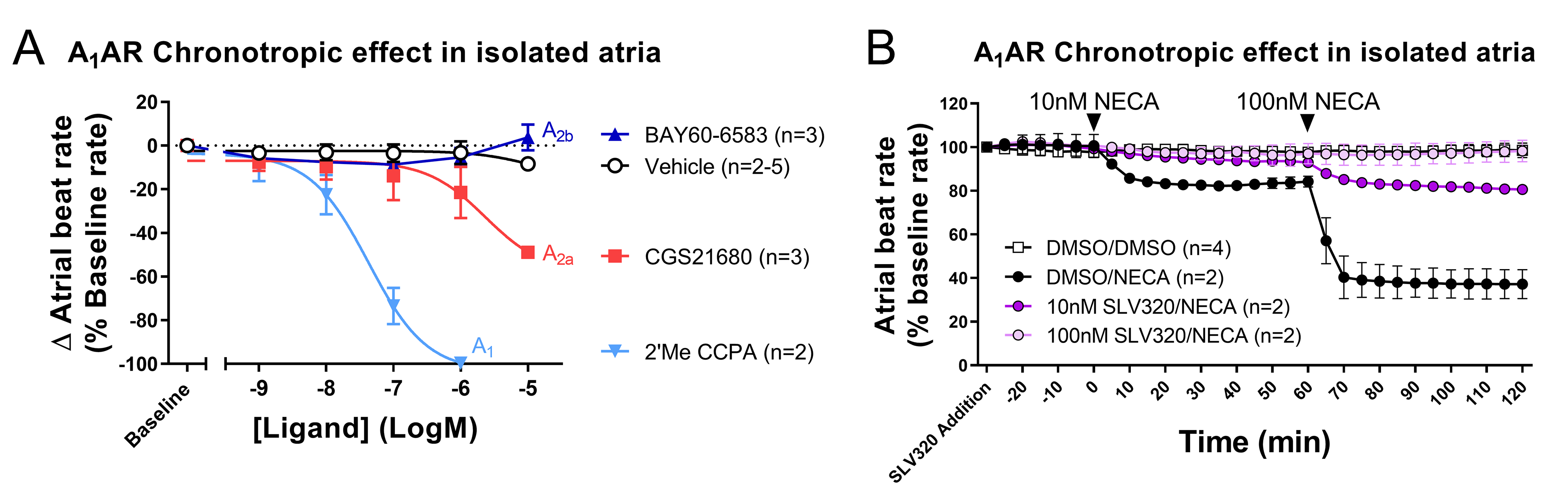


**Supp Figure 4**. Ex vivo chronotropic effects are A_1_R-mediated. **(A)** In isolated atria, the effect of NECA was recapitulated with the A_1_R agonist 2’Me CCPA, and to a lesser extent the A_2A_R-selective agonist CGS21680 (although this may be via activity at A_1_R). **(B)** Pretreatment with the A_1_R antagonist SLV320 inhibits NECA negative chronotropic response in isolated atria in a concentration-dependent manner. Data are displayed as mean ± SD.


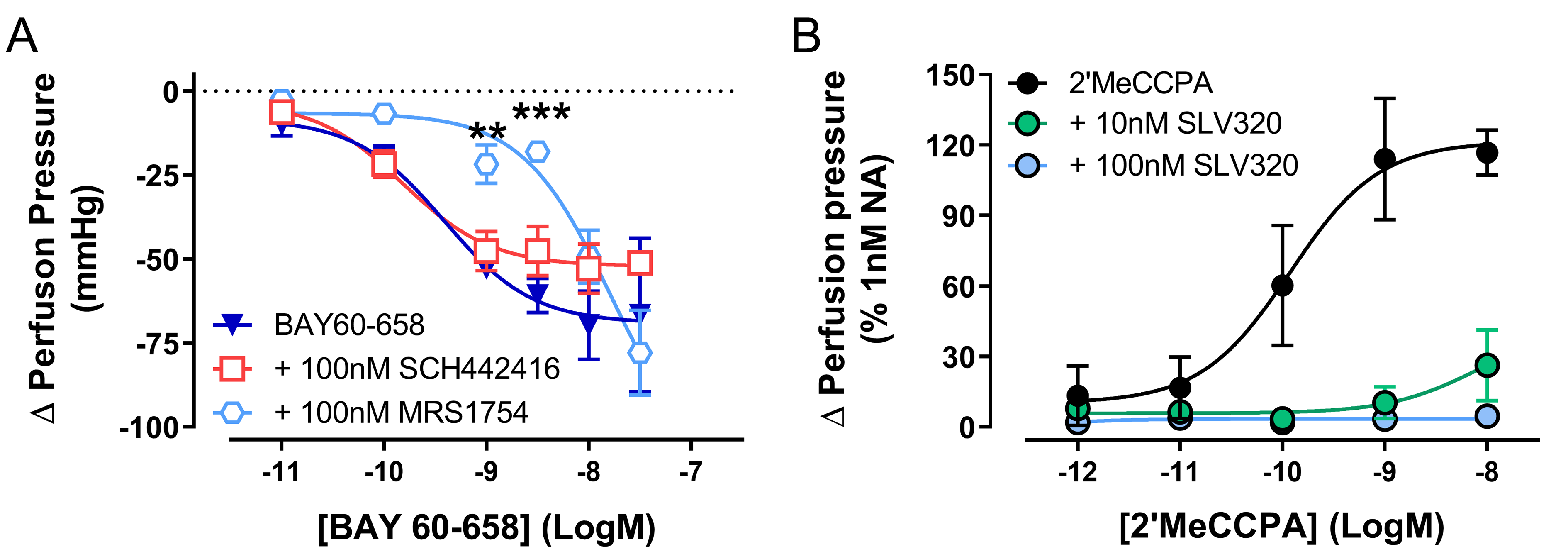


**Supp Figure 5**. Ex vivo renal vasodilation or vasoconstriction are A_2B_R- or A_1_R-mediated, respectively. (A) The A_2B_R-agonist BAY 60-658 (n=3) reduced perfusion pressure, which was inhibited by pretreatment with the A_2B_R-selective antagonist MRS1754 (n=3; two-way ANOVA, Dunnett’s post-test), but not the A_2A_R-selective antagonist SCH442416 (n=3). Data are displayed as mean ± SEM. 2’MeCCPA (n=3) promoted an increase in perfusion pressure (B), which was entirely A_1_R-mediated as it was concentration-dependently inhibited by pretreatment with the A_1_R-selective antagonist SLV320 at 10nM (n=4), and 100nM (n=2). Data are displayed as mean ± SD.


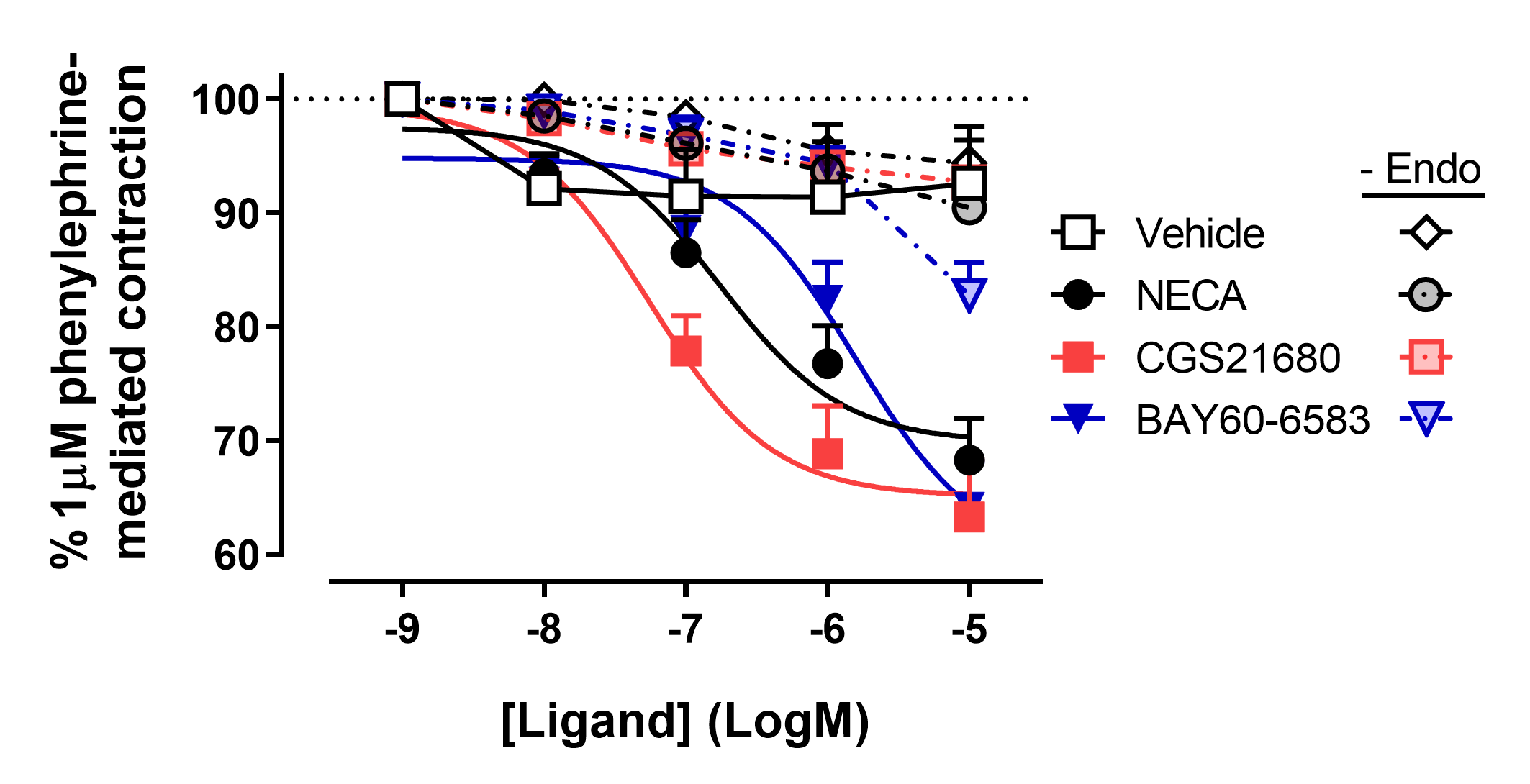


**Supp Figure 6**. *Ex vivo* rat thoracic aorta relaxation effects are dependent on endothelium and occur through activation of A_2A_R and A_2B_R. (A) Like NECA (pEC_50_ 6.8±0.2, n=9) the A_2A_R-selective agonist CGS21680 (pEC_50_ 7.3±0.2, n=8) and the A_2B_R-selective agonist BAY 60-6583 (pEC_50_ 5.8±0.2, n=10) produced concentration-dependent inhibition of phenylephrine-mediated aorta relaxation. Removing the endothelium completely abrogated the response to both CGS21680 (n=8) and BAY60-6583 (n=5), indicating this A_2_R-mediated relaxation effect is an endothelium-dependent mechanism. Data are expressed as mean ± SEM.
